# ENPP1 is an innate immune checkpoint of the anticancer cGAMP–STING pathway in breast cancer

**DOI:** 10.1073/pnas.2313693120

**Published:** 2023-12-20

**Authors:** Songnan Wang, Volker Böhnert, Alby J. Joseph, Valentino Sudaryo, Gemini Skariah, Jason T. Swinderman, Feiqiao B. Yu, Vishvak Subramanyam, Denise M. Wolf, Xuchao Lyu, Luke A. Gilbert, Laura J. van’t Veer, Hani Goodarzi, Lingyin Li

**Affiliations:** ^a^Department of Biochemistry, Stanford University, Stanford, CA 94305; ^b^ChEM-H Institute, Stanford University, Stanford, CA 94305; ^c^Arc Institute, Palo Alto, CA 94304; ^d^Department of Urology, University of California, San Francisco, CA 94143; ^e^Helen Diller Family Comprehensive Cancer Center, University of California, San Francisco, CA 94158; ^f^Department of Biophysics & Biochemistry, University of California, San Francisco, CA 94143; ^g^Baker Computational Health Science Institute, University of California, San Francisco, CA 94143; ^h^Department of Laboratory Medicine, University of California, San Francisco, CA 94115; ^i^Department of Pathology, Stanford University School of Medicine, Stanford, CA 94305

**Keywords:** ENPP1, extracellular cGAMP, STING, immune checkpoint, breast cancer metastasis

## Abstract

Ectonucleotide pyrophosphatase/phosphodiesterase 1 (ENPP1) expression correlates with poor prognosis in many cancers, and we previously discovered that ENPP1 is the dominant hydrolase of extracellular cGAMP: a cancer-cell-produced immunotransmitter that activates the anticancer stimulator of interferon genes (STING) pathway. However, ENPP1 has other catalytic activities and the molecular and cellular mechanisms contributing to its tumorigenic effects remain unclear. Here, using single-cell RNA-seq, we show that ENPP1 in both cancer and normal tissues drives primary breast tumor growth and metastasis by dampening extracellular 2′3′-cyclic-GMP-AMP (cGAMP)–STING-mediated antitumoral immunity. ENPP1 loss-of-function in both cancer cells and normal tissues slowed primary tumor growth and abolished metastasis. Selectively abolishing the cGAMP hydrolysis activity of ENPP1 phenocopied ENPP1 knockout in a STING-dependent manner, demonstrating that restoration of paracrine cGAMP–STING signaling is the dominant anti-cancer mechanism of ENPP1 inhibition. Finally, *ENPP1* expression in breast tumors deterministically predicated whether patients would remain free of distant metastasis after pembrolizumab (anti-PD-1) treatment followed by surgery. Altogether, ENPP1 blockade represents a strategy to exploit cancer-produced extracellular cGAMP for controlled local activation of STING and is therefore a promising therapeutic approach against breast cancer.

## Significance

Current immunotherapy is only effective toward patients with solid tumors infiltrated with immune cells or “hot” tumors. Ectonucleotide pyrophosphatase/phosphodiesterase 1 (ENPP1) expression has been correlated with poor cancer prognosis with unclear mechanisms. Our previous work uncovered ENPP1 as a negative regulator of the innate immune stimulator of interferon genes (STING) pathway by degrading extracellular 2′3′-cyclic-GMP-AMP (cGAMP). Here, we found that a mouse harboring a point-mutation in ENPP1 rendering it unable to degrade cGAMP is resistant to breast cancer metastasis in a STING-dependent manner. Breast cancer patients with low ENPP1 expression have hot tumors and responded completely to pembrolizumab with 7-y metastasis-free survival, demonstrating ENPP1 levels can be used as a biomarker for patient stratification and should also be targeted for immunotherapy.

The strategy of blocking adaptive immune checkpoints including programmed cell death protein 1 (PD-1), programmed death-ligand 1 (PD-L1), and cytotoxic T-lymphocyte associated protein 4 (CTLA-4) offers curative immunotherapy for some patients with otherwise terminal cancer diagnoses — however, only a minority of patients respond to immune checkpoint blockade (ICB) therapy and many cancer types remain inaccessible by this treatment (1–6). ICB resistance can occur through a variety of mechanisms, one of which is insufficient lymphocyte infiltration into the tumor, which is determined by our innate immune system’s ability to detect and communicate the presence of cancer (7–9). Cancer cells have a variety of strategies for suppressing innate immunity (10–13); therefore, a deep understanding of innate immune checkpoints holds the potential to unlock the full power of immunotherapy against immunologically “cold” tumors.

The cytosolic double-stranded DNA (dsDNA)-sensing stimulator of interferon genes (STING) pathway is a key innate immune pathway that detects and responds to chromosomal instability (CIN) and extrachromosomal DNA present in cancer cells (14–17). Cytosolic dsDNA is detected by the enzyme cyclic-GMP-AMP synthase (cGAS) (18), which synthesizes the cyclic dinucleotide 2′3′-cyclic-GMP-AMP (cGAMP) (19, 20). cGAMP binds and activates STING, leading to production of type I interferons (IFN-I) and downstream immune cell infiltration (21, 22). We and others discovered that cancer cells produce and secrete cGAMP into the extracellular space (23, 24), which is then taken up by surrounding host cells, leading to paracrine STING activation (25–29). We found that extracellular cGAMP is important for the curative effect of ionizing radiation (IR) in a murine breast cancer model (23). This serves as a hint of the importance of the extracellular cGAMP–STING axis in cancer. However, the extent to which extracellular cGAMP–STING controls antitumor immunity is unknown.

One important negative regulator of the extracellular cGAMP–STING pathway is ectonucleotide pyrophosphatase/phosphodiesterase 1 (ENPP1), the dominant hydrolase that degrades extracellular cGAMP (23, 30). There is mounting evidence that ENPP1 promotes breast cancer in humans (31, 32). However, it is unclear whether ENPP1 does so by inhibiting extracellular cGAMP–STING signaling or by affecting the biology of its other substrates including adenosine triphosphate (ATP). Our previous work showed that ENPP1 inhibitors have efficacy in murine models of primary breast cancers with limited effect size (23, 33). Therefore, it is also unclear whether mouse models would recapitulate human cancer biology.

In this study, we utilized a mouse model system where we observed impressive anti-metastatic effect when we removed ENPP1’s cGAMP hydrolysis activity that mirrors the survival advantage in patients with low *ENPP1*–expressing breast tumors. We determined that ENPP1 expression is an on/off switch that controls whether breast cancer will metastasize in a STING-dependent manner in mouse models. In addition, low *ENPP1* expression in pre-treatment biopsy samples obtained from breast cancer patients predicted their response to pembrolizumab (anti-PD-1) as a neoadjuvant therapy and distant-metastasis free survival (DMFS) up to 7 y. Together, we conclude that ENPP1 is an innate immune checkpoint restraining STING pathway activation in the tumor microenvironment (TME) and that therapeutically targeting ENPP1’s cGAMP hydrolysis activity could dramatically increase the number of patients who completely respond to anti-PD-1 therapies.

## Results

### ENPP1’s Catalytic Activity Drives Breast Tumor Growth and Metastasis by Restricting Adaptive Immune Infiltration.

**Fig. 1.**
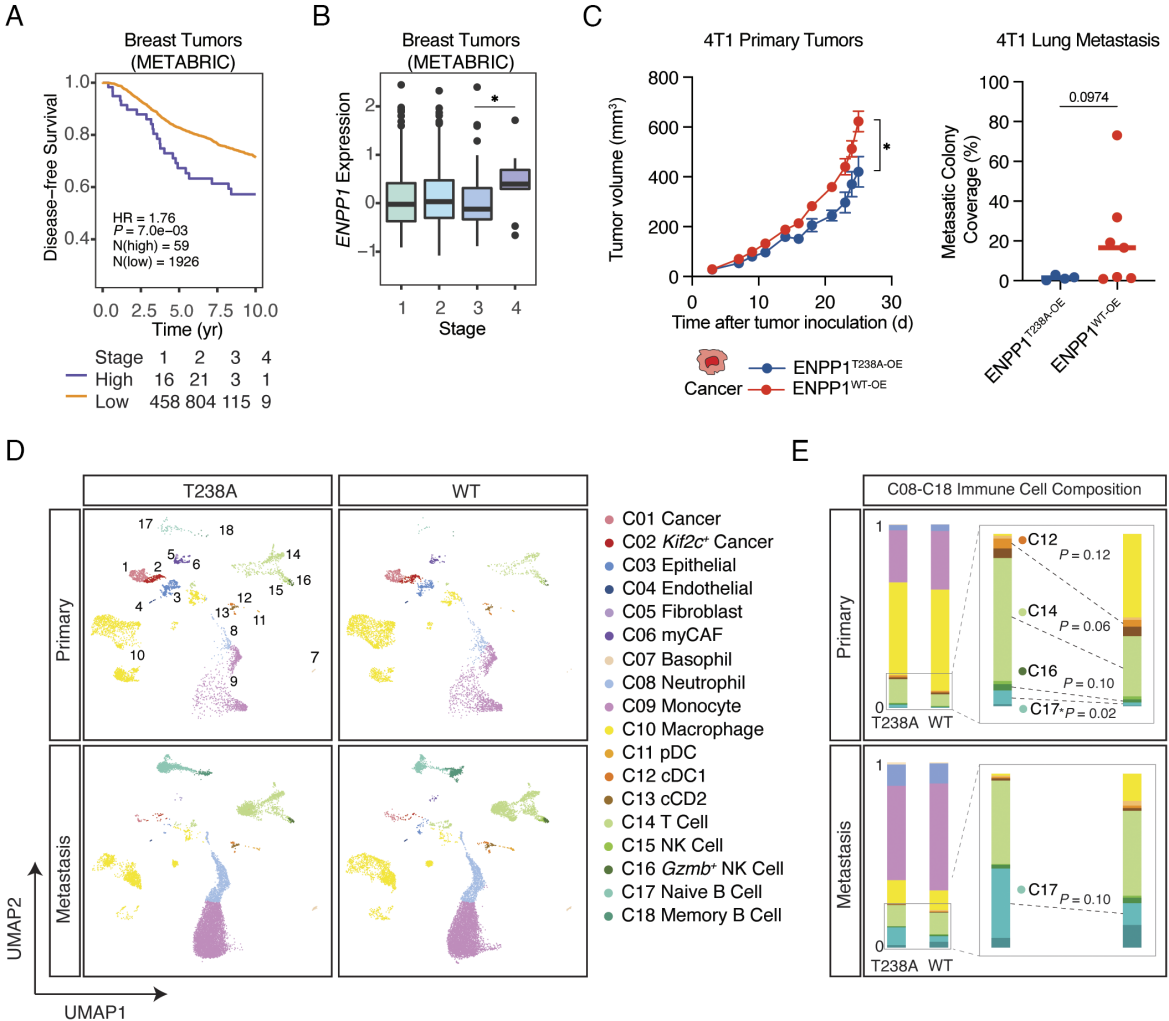
ENPP1’s catalytic activity drives breast tumor growth and metastasis by restricting immune infiltration. (*A*) Disease-free survival of breast cancer patients in the METABRIC database in the *ENPP1*-high group (n = 59) and *ENPP1*-low group (n = 1,926) and the number of patients stratified by stages. Threshold for high vs. low expression was set at which *P* value was the smallest. (*B*) *ENPP1* expression in patients with stage 1 to 4 breast cancer. Shown as box plots of median and interquartile levels. The *P* value was determined by the nonparametric Mann-Whitney *U* test. (*C*) Primary tumor volumes and quantification of lung metastases of WT BALB/cJ mice bearing ENPP1^T238A-OE^ and ENPP1^WT-OE^ 4T1 tumors (n = 5 and 9 mice). Tumor growth curves were plotted as mean ± SEM. Metastasis data were plotted as mean. *P* values were determined by the unpaired *t* test with Welch correction. (*D*) UMAP plots of the annotated clusters of ENPP1^T238A-OE^ and ENPP1^WT-OE^ 4T1 primary tumors and metastasis colonized lungs. (*E*) Barplots comparing immune cell compositions (containing C08-C18) between ENPP1^T238A-OE^ and ENPP1^WT-OE^ 4T1 primary tumors and metastasis colonized lungs. *P ≤ 0.05; *P* value is shown if it is between 0.05 and 0.15. See also *SI Appendix*, Figs. S1 and S2.

ENPP1 expression levels have been shown to correlate with poor prognosis in several cancer types. Patients in the METABRIC database with breast tumors expressing high levels of *ENPP1* mRNA have a significantly worse disease-free survival rate, despite exhibiting a similar distribution across disease stages as the *ENPP1*-low group (Fig. 1*A*). Furthermore, patients with stage IV metastatic disease have significantly higher *ENPP1* RNA expression than patients with stage III disease (Fig. 1*B*). To determine whether ENPP1 expression is causally linked to poor prognosis in breast cancer, we sought to perform mechanistic studies in mouse models. We performed orthotopic implantation of *Enpp1*-knockout 4T1 murine breast cancer cells overexpressing either WT ENPP1 (ENPP1^WT-OE^) or catalytically dead ENPP1 (ENPP1^T238A-OE^) into WT mice (*SI Appendix*, Fig. S1 *A*–*C*). ENPP1^WT-OE^ 4T1 cells exhibited faster primary tumor growth and more lung metastases (Fig. 1*C* and *SI Appendix*, Fig. S1 *E* and *F*) without affecting cell proliferation (*SI Appendix*, Fig. S1*D*), implicating a non-tumor cell-intrinsic mechanism of enhanced tumor growth and metastasis. To thoroughly characterize the impact of ENPP1 overexpression on the TME, we performed single-cell RNA-seq (scRNA-seq) on primary tumors and lungs colonized by metastases collected from this experiment. We observed 32,539 cells that passed quality filters. We performed unsupervised graph-based clustering on all cells and identified 18 major clusters corresponding to reported cell types by manual annotation of lineage markers. (Fig. 1*D* and *SI Appendix*, Fig. S2 *A* and *B*).

Although the ENPP1^WT-OE^ condition did not alter the composition of non-immune cells (*SI Appendix*, Fig. S2*C*), it led to a decreased proportion of conventional DC type 1 (cDC1s), T cells, and *Gzmb*^+^ cytotoxic NK cells in primary tumors (Fig. 1*E*). Unexpectedly, overexpression of WT ENPP1 resulted in a dramatic decrease in tumor-infiltrating naive B cells in both the primary and metastatic TME compared with the catalytic mutant (Fig. 1*E*). Together, we conclude that ENPP1’s catalytic activity restricts adaptive immune cell infiltration, contributing to its role in promoting breast cancer growth and metastasis.

### ENPP1’s Catalytic Activity Promotes Immune Suppression in Primary Tumors and Lung Metastases.

**Fig. 2.**
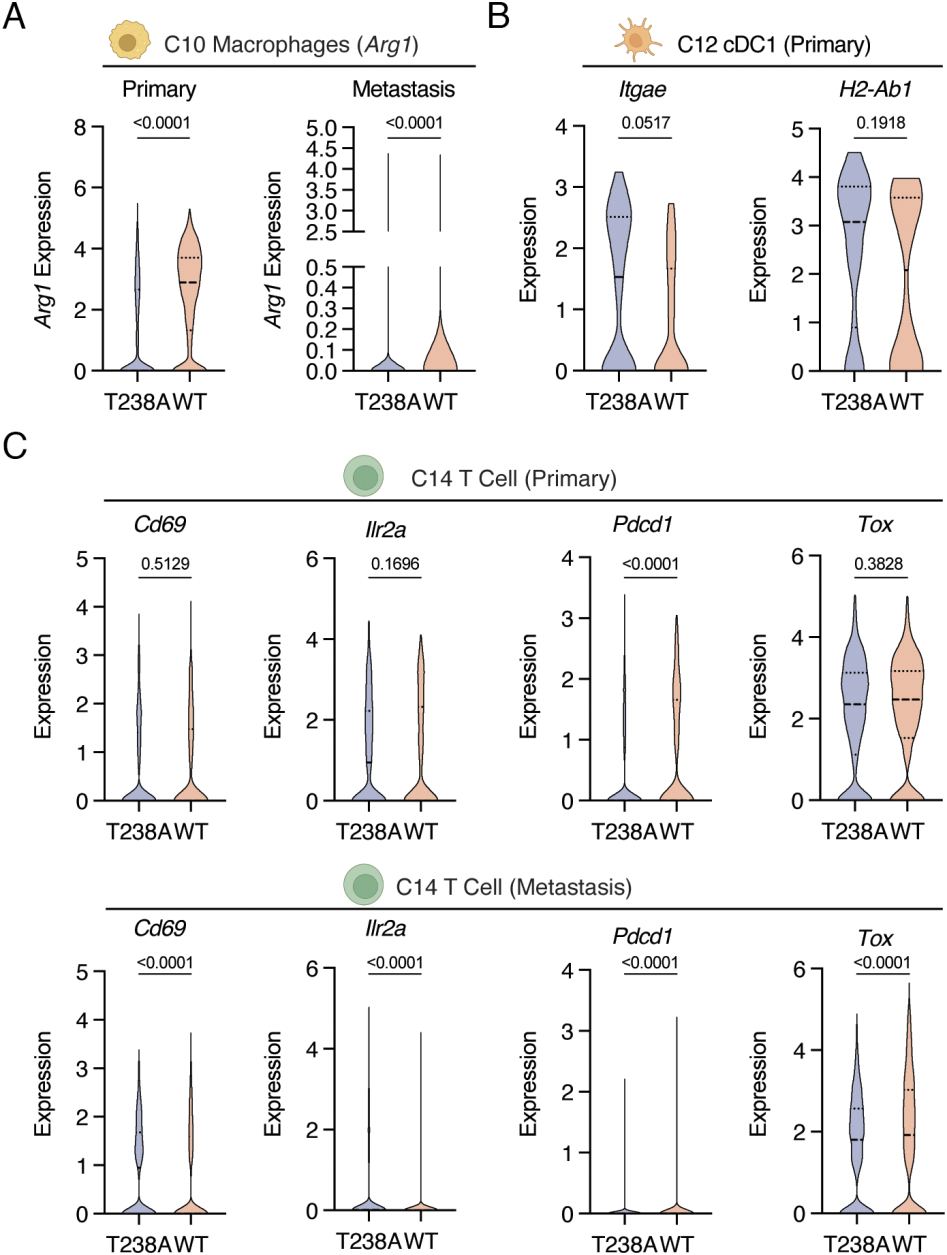
ENPP1’s catalytic activity promotes immune suppression in primary tumors and lung metastases. (*A*–*C*) Violin plots of indicated transcripts in indicated cell types comparing between ENPP1^T238A-OE^ and ENPP1^WT-OE^ 4T1 tumors or metastases. *Arg1* in macrophages in primary tumors and lung metastases (*A*); *Itgae* and *H2-Ab1* in cDC1s in primary tumors (*B*); *Cd69*, *Ilr2a*, *Pdcd1*, and *Tox* in T cells in primary tumors and lung metastases (*C*). *P* values were determined by the nonparametric Mann-Whitney *U* test. cDC1 stands for conventional dendritic cell type 1. See also *SI Appendix*, Figs. S2 and S3.

Building on our findings that ENPP1 catalytic activity alters the composition of the tumor immune compartment, we next investigated its impact on the functional landscape of tumor-infiltrating immune cells. We first turned our attention to innate immune cells. We found increased expression of Arginase 1 (*Arg1*) in monocytes and macrophages in mice injected with ENPP1^WT-OE^ 4T1 cells (Fig. 2*A* and *SI Appendix*, Fig. S2*D*), which is associated with pro-tumor myeloid-derived suppressor cells (MDSCs) (34) and M2-like macrophages (35), respectively. We further categorized macrophages into four subtypes based on expression of reported macrophage identity markers (36) and functional markers (*SI Appendix*, Fig. S3 *A* and *B*). These four tumor-associated macrophage (TAM) subsets express different levels of anti-tumor M1-like macrophage markers vs. pro-tumor M2-like macrophage markers, with *Vcan*^+^ TAMs being the most M1-like, and *Pparg^+^* being exclusively immunosuppressive (*SI Appendix*, Fig. S3*B*). We observed a decrease in *Vcan*^+^ and *C1qc^+^* TAMs and a concomitant increase in M2-like *Spp1^+^* TAMs in ENPP1^WT-OE^, suggesting that ENPP1 catalytic activity favors the polarization of M1-like to M2-like TAMs (*SI Appendix*, Fig. S3*C*). This is consistent with our previously reported effect of extracellular cGAMP depletion on macrophage polarization (29).

Focusing on antigen-presenting cells (APCs) that orchestrate innate-adaptive crosstalk, we noticed a decrease in *Itgae* expressing migratory cDC1 in primary tumors, but not metastases, in the presence of ENPP1 catalytic activity (Fig. 2*B* and *SI Appendix*, Fig. S2*E*), mirroring the effects we previously observed with extracellular cGAMP depletion during IR treatment (23). Additionally, cDC1s in ENPP1^WT-OE^ primary tumors, but not metastases, express less *H2-Ab1*, suggesting decreased antigen-presentation capacity (Fig. 2*B* and *SI Appendix*, Fig. S2*E*). We reason that APC recruitment and education at the initial site of cancer encounter is important in mounting a successful adaptive immune response, and this process appears to be diminished when tumors overexpress ENPP1.

Examining changes in adaptive immune cells, we found that the relative abundance of T cell subpopulations was not significantly altered between the WT and catalytic mutant ENPP1 conditions (*SI Appendix*, Fig. S3 *D*–*F*). However, compared to ENPP1^T238A-OE^, WT ENPP1 activity decreased the expression of *Cd69* (an early T cell activation marker) and *Ilr2a* (a late T cell activation marker), and increased the expression of *Pdcd1* and *Tox* (exhausted T cell markers) in the T cells infiltrating both primary tumors and lung metastases (Fig. 2*C*). In summary, our scRNA-seq data suggest that cancer-cell-derived ENPP1 catalytic activity attenuated T cell activation while promoting exhaustion in primary tumors and sites of metastasis. Taken together, ENPP1 catalytic activity shapes the immunosuppressive TME both in primary tumors and metastases.

### ENPP1 Overexpression in Cancer Cells Inhibits STING Signaling to Suppress Anti-Tumor Immunity.

**Fig. 3.**
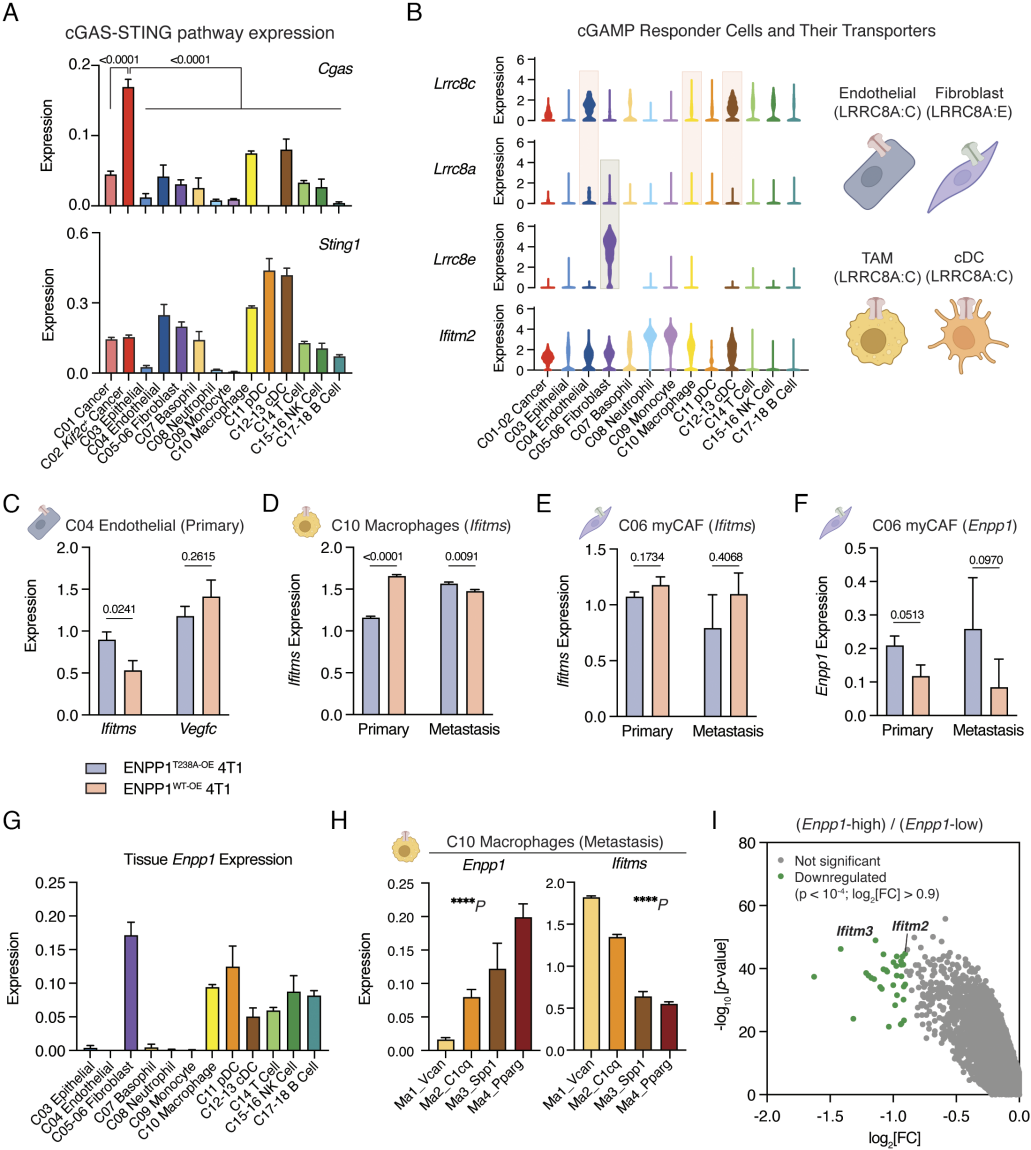
ENPP1 expressed on cancer and responder cells blocks paracrine cGAMP–STING activation. (*A*) Bar graphs of *Cgas* and *Sting1* expression across the annotated clusters. *P* values were determined by the nonparametric Mann-Whitney *U* test. (*B*) Violin plots of *Lrrc8c*, *Lrrc8a*, *Lrrc8e*, and *Ifitm2* across the annotated clusters. Schematic of cGAMP responder cells and their putative transporters: LRRC8A:C in endothelial cells, TAM and cDC; LRRC8A:E in fibroblasts. (*C*–*F*) Bar graphs of indicated transcripts in indicated cell types comparing between ENPP1^T238A-OE^ and ENPP1^WT-OE^ 4T1 tumors or metastases. *Ifitms* and *Vegfc* in endothelial cells in primary tumors (*C*); *Ifitms* in macrophages in primary tumors and metastases (*D*); *Ifitms* in myCAFs in primary tumors and metastases (*E*); *Enpp1* in myCAFs in primary tumors and metastases (*F*). *P* values in *C* and *D* were determined by the nonparametric Mann-Whitney *U* test. (*G*) Bar graphs of *Enpp1* expression across the annotated clusters. (*H*) Bar graphs of *Enpp1* and *Ifitms* across the annotated macrophage subclusters. *P* values were determined by the ordinary one-way ANOVA test. (*I*) Differentially expressed genes in *Enpp1*-high vs*. Enpp1*-low groups. Bars represent mean ± SEM. TAM stands for TAMs. cDC stands for conventional dendritic cell, combining both cDC1 and cDC2. myCAF stands for myofibroblastic cancer-associated fibroblast. See also *SI Appendix*, Figs. S4 and S5.

To understand the mechanisms of cancer-derived ENPP1 overexpression in driving immunosuppression, we first analyzed expression of genes in the extracellular cGAMP–STING pathway (Fig. 3 *A* and *B* and *SI Appendix*, Fig. S4*A*). Notably, *Cgas* expression is the highest in a distinct cancer cell cluster annotated for overexpressing *Kif2c*, which has been reported to drive CIN and metastasis (17). In contrast, *Sting1* is expressed at relatively high levels in endothelial cells, fibroblasts, macrophages, and DCs (Fig. 3*A*). The differential expression of cGAS and STING supports our model that cancers produce and secrete cGAMP, which is then detected by surrounding host cells (23). We defined potential cGAMP responder cells as those with low expression of *Cgas* and high expression of *Sting1* and interferon-stimulated genes (ISGs) *Ifitm1*, *Ifitm2*, and *Ifitm3* (Fig. 3*B* and *SI Appendix*, Fig. S4*A*), which matched well with our previous report of cell types that respond to extracellular cGAMP (29). We then examined expression of the only known murine cGAMP transporters, LRRC8A:C and LRRC8A:E complexes (27, 28), in these responder cells. While endothelial cells, macrophages, and cDCs express genes encoding the LRRC8A:C complex, fibroblasts uniquely express genes encoding the LRRC8A:E complex (Fig. 3*B*).

We next examined how overexpression of WT ENPP1 in cancer cells affected STING activation in cGAMP responder cells as measured by their combined *Ifitm1*, *Ifitm2*, and *Ifitm3* expression (*Ifitms*). Looking first at endothelial cells, we found that elevated ENPP1 catalytic activity from ENPP1^WT-OE^ 4T1s suppressed endothelial *Ifitms* expression, with a more pronounced effect in primary tumors than lung metastases (Fig. 3*C* and *SI Appendix*, Fig. S4*B*). IFN signaling is known to downregulate vascular endothelial growth factors (VEGFs) and inhibit angiogenesis (37); we observed that endothelial cells with blunted IFN signaling in ENPP1^WT-OE^ primary tumors express more *Vegfc* (Fig. 3*C* and *SI Appendix*, Fig. S4*B*). Turning our focus to TAMs, we show that in metastasis, ENPP1^WT-OE^ 4T1s with increased cGAMP degradation activity led to decreased expression of *Ifitms* as expected (Fig. 3*D*). However, we observed the opposite trend in primary tumors where *Ifitms* expression in TAMs increased in ENPP1^WT-OE^ tumors (Fig. 3*D*). We noticed that *Lrrc8c* expression in TAMs is also higher in ENPP1^WT-OE^ primary tumors but not metastases (*SI Appendix*, Fig. S4*C*). It is possible that increased cGAMP import activity in ENPP1^WT-OE^ primary tumors contributes to the unexpected increase in their *Ifitms* expression.

To understand how ENPP1^WT-OE^ 4T1s promote immunosuppressive phenotypes in primary tumor-resident TAMs despite increased ISG expression in these cells, we examined the activation status of the extracellular adenosine (eADO) pathway: a potential STING-independent downstream effect of cGAMP hydrolysis (*SI Appendix*, Fig. S5*A*). We observed increased expression of *Adora2b* (the eADO receptor), *Tgfb1* and *Il10rb* (downstream of eADO signaling), and *Hp* (Haptoglobin) (a gene that is transcriptionally upregulated by adenosine signaling) in ENPP1^WT-OE^ primary TAMs, indicating activation of the eADO pathway in TAMs by ENPP1 catalytic activity specifically in primary tumors but not in metastases (*SI Appendix*, Fig. S5*B*) (38–40). HP secretion by cancer cells upon of eADO signaling has been reported to recruit polymorphonuclear MDSCs and promote self-seeding of ENPP1-high circulating tumor cells (40). While we did not observe increased *Hp* expression in ENPP1^WT-OE^ cancer cells, we found that neutrophils and monocytes in the ENPP1^WT-OE^ metastatic niche had the biggest increase and the highest overall expression of *Hp*, suggesting that they are the potential source of HP production that facilitate metastasis (*SI Appendix*, Fig. S5*C*). Together, our data suggest a model in which ENPP1 overexpression promotes primary tumor growth and metastasis through synergistic stimulation of the eADO pathway and inhibition of the STING pathway (*SI Appendix*, Fig. S5*D*).

### ENPP1 Expressed on Host Responder Cells Suppresses Paracrine STING Activation.

We next turned our attention to a third class of cGAMP responder cells, myofibroblast-like cancer-associated fibroblasts (myCAFs): a subtype of fibroblasts with immunosuppressive functions (41). While the results were not statistically significant due to small sample sizes, we noticed that myCAFs, similar to TAMs, trended toward a decrease in STING pathway activation as measured by *Ifitms* expression, with a concomitant increase in *Enpp1* expression, in ENPP1^T238A-OE^ primary tumors (Fig. 3 *E* and *F*). We observed similar increases in *ENPP1* expression along the oncogenic trajectory from normal tissue to tumor-adjacent tissue to primary tumor in human breast invasive carcinoma (BRCA) (*SI Appendix*, Fig. S4*D*). Previous studies and our analysis so far have focused on ENPP1 expressed by cancer cells. However, these data hint at the importance of the ENPP1 level expressed by a responder cell in regulating its paracrine STING signaling.

To build a more comprehensive picture of *Enpp1* expression across host cells in the TME, we examined its expression level and downstream impact on STING activation in stromal and immune cells. Among the responder cells, we found that fibroblasts, macrophages, and DCs express relatively high levels of *Enpp1* (Fig. 3*G*). Endothelial cells, on the other hand, do not express measurable levels of *Enpp1* (Fig. 3*G*), which could explain the predominant effect of cancer-derived ENPP1 on their STING activation profile (Fig. 3*C*). Additionally, we found that *Ifitms* expression in subpopulations of TAMs in the metastases anti-correlates with their *Enpp1* expression level (Fig. 3*H*). Specifically, there is a step-wise increase in *Enpp1* expression and a corresponding decrease in *Ifitms* expression from the immunostimulatory M1-like macrophages to the immunosuppressive M2-like macrophages (Fig. 3*H*). This result could potentially explain our previous findings that M2-polarized macrophages are less sensitive to cancer-derived extracellular cGAMP than M1-polarized macrophages (29).

Expanding this correlation to other cell types, we performed differential gene expression analysis between *Enpp1*-high cells (Enpp1 > 1.24, 338 cells) and *Enpp1*-low cells (0 < *Enpp1* < 1.24, 1,040 cells) and found that *Ifitm2* and *Ifitm3* are among the most significantly downregulated genes in *Enpp1*-high cells (Fig. 3*I*). Together, our results demonstrate that ENPP1 expressed on cGAMP responder cells potently inhibits these cells’ paracrine STING activation. These findings suggest the need to inhibit both cancer- and host-derived ENPP1 to alleviate its tumorigenic effect and emphasize the impact of host ENPP1 expression level in different cell types on the downstream effects of paracrine STING signaling.

### *Enpp1* Knockout in Cancer and Tissue Cells Additively Delays Tumor Growth and Abolishes Metastasis.

**Fig. 4.**
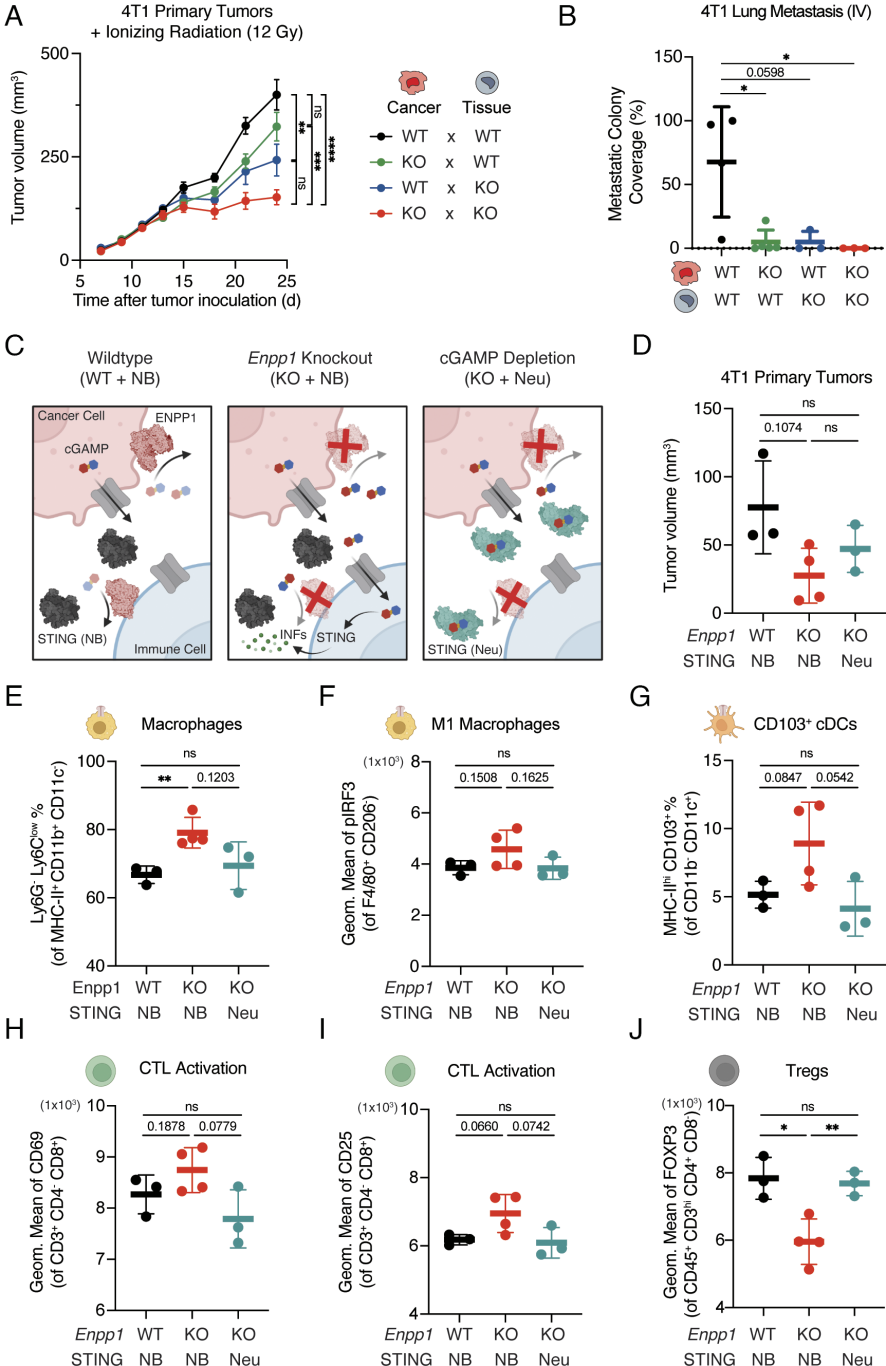
The antitumoral and immunostimulatory effect of ENPP1 deficiency is connected to extracellular cGAMP levels. (*A*) Primary tumor volumes of WT or *Enpp1*^−/−^ 4T1 BALB/cJ mice orthotopically injected with WT or *Enpp1*^−/−^ 4T1 (n = 7, 17, 9, 16 mice for *Enpp1* KO × KO, KO × WT, WT × KO, and WT × WT cancer × tissue genotype combinations). Data were plotted as mean ± SEM. *P* values of the last tumor measurement were determined by the multiple unpaired *t* test with Welch correction. (*B*) Quantifications of lung metastatic colonies of WT or *Enpp1*^−/−^ 4T1 BALB/cJ mice intravenously injected with WT or *Enpp1*^−/−^ 4T1 (n = 4, 5, 3, 3 mice for *Enpp1* KO × KO, KO × WT, WT × KO, and WT × WT cancer × tissue genotype combinations). Data were plotted as mean ± SD. *P* values were determined by the unpaired *t* test. (*C*) Schematic of experimental strategies of comparing between wildtype, *Enpp1* knockout, and extracellular cGAMP depletion through genetic manipulation and cGAMP neutralization. Structures of dimer ENPP1 (PWD: 4B56), monomer ENPP1 (PWD: 6XKD), mSTING (PWD: 4KCO), and mSTING bound with DMXAA (PWD: 4LOL). (*D*) Primary tumor volumes of *Enpp1* WT mice receiving *Enpp1* WT 4T1 and R237A non-binding STING injection (WT + NB) (n = 3 biological replicates), *Enpp1* KO mice receiving *Enpp1* KO 4T1 and NB STING injection (KO + NB) (n = 4 biological replicates), and *Enpp1* KO mice receiving *Enpp1* KO 4T1 and WT neutralizing STING injection (KO + Neu) (n = 3 biological replicates). (*E*) The percentage of Ly6G^−^Ly6C^low^ cells out of MHC-II^+^CD11b^+^CD11c^−^ macrophages. (*F*) The geometric mean of pIRF3 of F40/80^+^CD206^−^ M1-like macrophages. (*G*) The percentage of MHC-II^hi^CD103^+^ cells out of CD11b^-^CD11c^+^ cells. (*H* and *I*) The geometric mean of CD69 (*H*) and CD25 (*I*) of CD3^+^CD4^−^CD8^+^ cytotoxic T lymphocytes (CTLs). (*J*) The geometric mean of FOXP3 of CD45^+^CD3^hi^CD4^+^CD8^−^ regulatory T cells (Tregs). (*D* and *E*) Data were plotted as mean ± SD. *P* values were determined by the unpaired *t* test with Welch correction. *P ≤ 0.05., **P ≤ 0.01; *P* value is shown if between 0.05 and 0.2; not significant (ns). See also *SI Appendix*, Fig. S6.

Next, we formally tested the relative contribution of cancer- and host-derived ENPP1 on breast tumor growth and metastasis. We implanted WT or *Enpp1^−/−^* 4T1 cells into WT or *Enpp1^−/−^* BALB/cJ mice and treated established tumors (palpable around 100 mm^3^) with IR to further induce cGAMP production (23). Indeed, depleting cancer- or tissue-derived ENPP1 had an additive effect on slowing tumor growth, with tissue ENPP1 playing a larger role (Fig. 4*A*). Cancer- and host-derived ENPP1 contribute equally to intratumoral cGAMP degradation activity, while tissue-derived ENPP1 is mainly responsible for cGAMP degradation in serum (*SI Appendix*, Fig. S6*A*).

We also investigated how the ENPP1 source impacts metastasis by orthotopically implanting WT or *Enpp1^−/−^* 4T1 cells into WT or *Enpp1^−/−^* 4T1 mice, respectively (WT × WT vs. KO × KO), and collected various organs for ex vivo culturing at experimental end point. In WT × WT mice, distal metastasis was observed in the draining inguinal lymph node (dLN), blood, lung, and liver, but not in the brain. On the other hand, we observed no metastasis in KO × KO mice (*SI Appendix*, Fig. S6*B*). Since the KO × KO mice also have attenuated primary tumor growth, we reasoned that this could mask direct effects on metastasis, and therefore sought to disambiguate metastasis from primary tumor growth rate by intravenously injecting 4T1 cells. Again, we found that total loss of ENPP1 in cancer cells and host tissue rendered two thirds of the mice metastasis-free (Fig. 4*B* and *SI Appendix*, Fig. S6*C*). Our results support a model in which both tissue-derived and tumor-derived ENPP1 act in concert to promote primary tumor growth and distal organ metastasis. Importantly, the complete lack of detectable metastasis in most ENPP1-deficient animals harboring ENPP1-deficient tumors indicates a significant therapeutic potential of ENPP1 inhibition to protect against metastasis.

### The Antitumoral and Immunostimulatory Effect of ENPP1 Deficiency Is Connected to Extracellular cGAMP Levels.

Our scRNA-seq analysis suggested that both dampening STING signaling and promoting eADO signaling contribute to an immunosuppressive primary TME in ENPP1 overexpressing tumors. When reversed to consider the ENPP1-deficient condition, we hypothesize that as less eADO will be generated, the extracellular cGAMP–STING signaling will play a dominant role in immune recruitment and activation. To test this hypothesis, we took advantage of previously developed cell-impermeable STING protein as a neutralizing agent to deplete extracellular cGAMP and compared it with R237A mutant STING that does not bind to cGAMP as a negative control (23, 29). We injected either WT neutralizing STING (Neu) or R237A non-binding STING (NB) into WT mice bearing WT 4T1 orthotopic tumors (WT × WT) or *Enpp1* KO mice with ENPP1 KO 4T1 orthotopic tumors (KO × KO) (Fig. 4*C*). On day 15, we observed a near 75% reduction in average tumor sizes upon *Enpp1* deletion: an effect that was around 50% rescued with extracellular cGAMP neutralization but not with the NB STING treatment (Fig. 4*D*). This trend suggests that *Enpp1* knockout slows primary tumor growth at least partially through enhancing extracellular cGAMP levels.

We also analyzed the immunological changes in these primary tumors in response to *Enpp1* knockout with or without extracellular cGAMP depletion by flow cytometry. We observed an increase in the percentage of macrophages in KO × KO tumors, a trend that was reversed upon extracellular cGAMP depletion (Fig. 4*E*). In KO × KO tumors, we observed moderate increase in STING activation in M1-like macrophages measured by IRF3 phosphorylation (Fig. 4*F*), number of CD103^+^ migratory cDCs (Fig. 4*G*), and expression of activation markers CD69 and CD25 in cytotoxic T cells (Fig. 4 *H* and *I*). Additionally, we observed decreased immunosuppressive regulatory T cell (Treg) marker FOXP3, all correlating with a more immunostimulatory TME (Fig. 4*J*). However, these modest effects were abolished upon extracellular cGAMP depletion (Fig. 4 *F*–*J*). Together, our data draw a connection between enhanced extracellular cGAMP signaling and immunological control of primary tumor growth upon *Enpp1* loss.

### Selective Inhibition of ENPP1’s cGAMP Hydrolysis Activity Abolishes Breast Cancer Metastasis a STING-Dependent Manner.

**Fig. 5.**
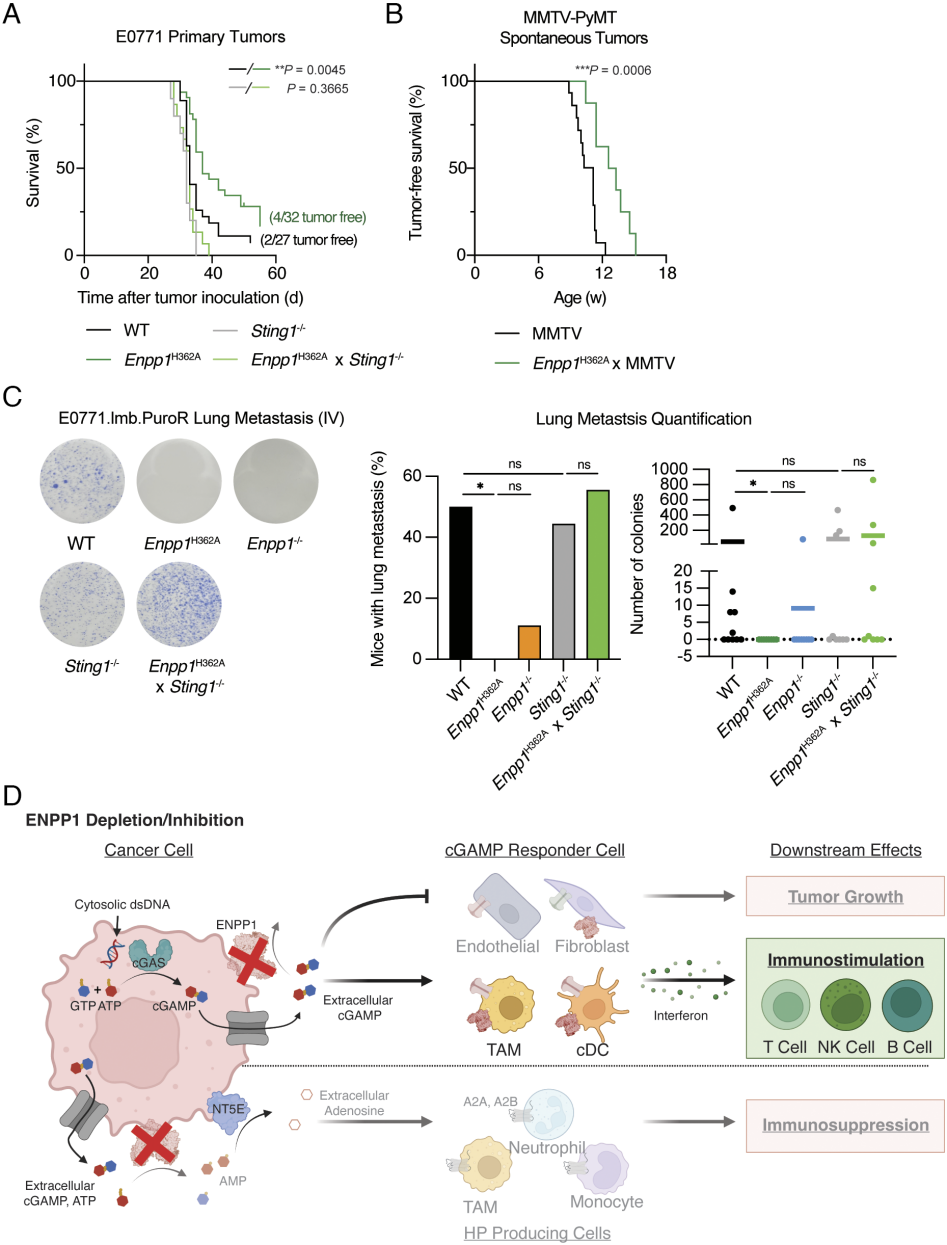
Selective inhibition of ENPP1’s cGAMP hydrolysis activity abolishes breast cancer metastasis in a STING-dependent manner. (*A*) Survival of WT, *Enpp1^H362A^, Sting1^−/−^*, and *Enpp1^H362A^* × *Sting1^−/−^* C57BL/6J mice (n = 27, 32, 10, 15 mice) bearing orthotopic E0771 breast tumors. Survival was measured by time taken for orthotopic E0771 breast tumors to reach 1,000 m^3^. (*B*) Tumor-free survival of MMTV and *Enpp1^H362A^* × MMTV mice (n = 15 and 18 mice) that developed spontaneous breast tumors. Tumor-free survival was measured by onset of the first spontaneous breast tumors. (*C*) Representative images and quantification of lung metastatic colonies of WT, *Enpp1^H362A^*, *Enpp1^−/−^. Sting1^−/−^* and *Enpp1^H362A^* × *Sting1^−/−^*C57BL/6J mice (*n* = 10, 8, 9, 9, 9 mice) intravenously injected with E0771.lmb.PuroR cells. Data are shown as mean. *P* values comparing the percentage of mice with lung metastasis were determined by the chi-squared test. *P* values comparing number of colonies were determined by the nonparametric Mann-Whitney *U* test. (*D*) Proposed model of mechanism of action in ENPP1 depletion/inhibition. *P* value for Kaplan–Meier curves were determined by the log-rank Mantel-Cox test. *P ≤ 0.05, **P ≤ 0.01, ***P ≤ 0.001. not significant (ns).

Apart from its cGAMP hydrolysis activity, ENPP1 is also known to degrade extracellular ATP (42) and generate immunomodulatory adenosine as byproduct (32). While our experiments above support a model in which extracellular cGAMP signaling is at least partially responsible for the effects of ENPP1 on tumorigenesis, we wanted to formally test the sufficiency of cGAMP hydrolysis to explain ENPP1’s pro-tumorigenic phenotypes. We took advantage of the previously developed homozygous *Enpp1^H362A^* mouse model: a separation-of-function point mutant that does not degrade cGAMP but retains its catalytic activity toward ATP and other nucleotide triphosphate substrates (42). Expanding beyond the 4T1 tumor model, we observed delayed primary E0771 tumor growth as measured by improved survival outcomes (time taken for tumors to reach 1,000 mm^3^) in *Enpp1^H362A^* mice compared to WT mice (Fig. 5*A*). *Enpp1^H362A^*mice retarded E0771 tumor growth to a similar degree as *Enpp1^−/−^* mice, as compared to WT mice (23) and the tumor slowing effects in *Enpp1^H362A^* mice were completely abolished in the *Sting1* knockout background (Fig. 5*A*).

Furthermore, we noticed a 41% increase in the tumor-free rate in *Enpp1^H362A^* compared with WT mice after E0771 implantation (4/32 [12.5%] vs. 2/27 [7.4%]) (Fig. 5*A*). Therefore, we hypothesized that *Enpp1* depletion also disfavors primary tumor onset. To test this hypothesis, we adopted a spontaneous breast tumor model of hemizygous mice harboring the mouse mammary tumor virus-polyoma middle tumor-antigen (MMTV-PyMT, MMTV for short). The median time for female MMTV mice to develop spontaneous breast tumors is 11 wk of age (Fig. 5*B*). Remarkably, *Enpp1*^H362A^ × MMTV mice with impaired extracellular cGAMP degradation activity exhibited delayed median tumor onset by 2 wk (Fig. 5*B*). These findings support cGAMP-mediated protection from tumor initiation upon ENPP1 blockade.

Last, to determine whether blocking ENPP1’s cGAMP hydrolysis activity alone is sufficient for preventing metastasis, we intravenously injected E0771.lmb.PuroR (a derived metastatic cell line from the parental E0771 cells (43) engineered with puromycin resistance to allow for ex vivo selection) into mice of different genetic backgrounds. Of note, 50% of WT mice but none of the *Enpp1^H362A^* mice and only 11% of *Enpp1^−/−^* mice had lung metastasis. Moreover, the anti-metastatic effect of blocking ENPP1’s cGAMP hydrolysis activity in the *Enpp1^H362A^* mice is completely STING-dependent (Fig. 5*C*). Together, we put forward a model that boosting extracellular cGAMP–STING signaling is the major mechanism of action of ENPP1 inhibition in mounting immune protection against breast cancer (Fig. 5*D*). While WT had slower primary tumor growth as *Sting1^−/−^*mice (Fig. 5*A*), the two genotypes had similar lung metastatic burden (Fig. 5*C*), indicating that endogenous ENPP1 activity in the tissue fully blocks STING-mediated immunological protection specifically against metastasis. Therefore, we postulate that deactivating ENPP1’s cGAMP hydrolysis activity to enhance paracrine STING signaling will be a promising therapeutic approach to impede breast cancer metastasis.

### *ENPP1* Expression Predicts Response and Prognosis of Breast Cancer Patients Receiving Anti-PD-1 Neoadjuvant Therapy.

**Fig. 6.**
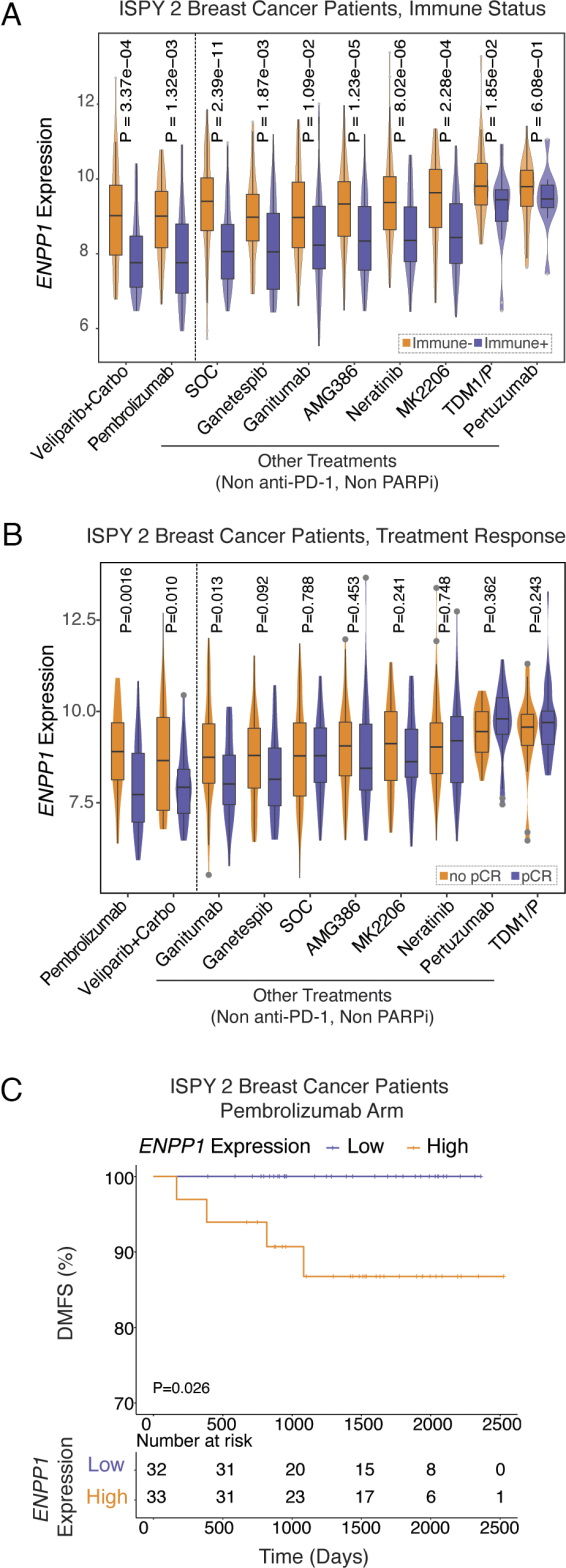
*ENPP1* expression predicts response and outcomes of breast cancer patients receiving anti-PD-1 neoadjuvant therapy. (*A* and *B*) *ENPP1* expression in Immune+ vs. immune- patients (*A*) and pCR vs. no pCR patients (*B*) across 10 treatment arms in the ISPY 2 Trial. *P* values were determined by the nonparametric Mann-Whitney *U* test. (*C*) DMFS of patients in the pembrolizumab arm in the ISPY 2 Trial with high *ENPP1* expression (n = 33) vs. low *ENPP1* expression (n = 32). *P* value was determined by the log-rank Mantel-Cox test. Immune+ stands for immune-positive; immune- stands for immune-negative; Carbo stands for Carboplatin; pCR stands for pathological complete response; DMFS stands for distant metastasis-free survival; HR stands for hazard ratio.

After delineating the molecular and cellular mechanisms governing the deterministic role of ENPP1 in metastasis of murine breast cancers, we asked whether these mechanistic insights and disease outcomes are conserved in humans. To this end, we analyzed the ISPY 2 breast cancer clinical trial data (44). Indeed, patients who had immune-positive (Immune+) tumors as defined by dendritic cell infiltration and STAT1 expression (downstream of IFN-I signaling) (45) had significantly less *ENPP1* mRNA expression universally across all 10 treatment arms (Fig. 6*A*). In addition, *ENPP1*-low patients had statistically significantly higher chance of pathological complete response (pCR) in Pembrolizumab (anti-PD-1) and Veliparib (poly-ADP ribose polymerase inhibitor, PARPi) + Carboplatin treatment groups as neoadjuvant therapies, but not in other treatment groups (Fig. 6*B*). These are the only two treatment arms out of the ten arms that have a clear mechanistic connection to cGAMP–STING signaling since anti-PD-1 efficacy requires CD8^+^ T cell infiltration (46) and PARPi induces DNA damage which increases cGAMP production (47). Most strikingly, 100% of *ENPP1*-low patients (n = 32) remained free of distant metastasis to date, near 7 y after Pembrolizumab treatment and surgery, while only around 85% of *ENPP1*-high patients (n = 33) remained free of distant metastasis. (Fig. 6*C*). Together, patients may have achieved complete response to Pembrolizumab at least partially due to enhanced cGAMP–STING-mediated immune infiltration permitted by an *ENPP1*-low setting, which confers long-term advantage in prognosis. The strong correlation suggests that lowering ENPP1 activity in humans is also sufficient to elicit cGAMP–STING signaling and provide long-term protection to metastasis. ENPP1 blockade is therefore a promising immuno-oncology strategy that could synergize with existing anti-PD-1/PD-L1 therapies in the clinics.

## Discussion

Motivated by a lack of understanding of the role of paracrine cGAMP–STING signaling in cancer and contradictory mechanistic hypotheses of ENPP1’s role in cancer immunity, we aimed to uncover the causal molecular and cellular mechanisms by which ENPP1 impacts primary breast tumor growth and metastasis. While we previously identified ENPP1 as a cGAMP hydrolase, there has been significant debate as to whether its ability to deplete cGAMP and thereby dampen STING signaling is central to its pro-tumorigenic effects, as ENPP1 has other enzymatic activities toward ATP and other nucleotide triphosphates but also generates eADO—a cancer-associated metabolite—as a byproduct of its cGAMP hydrolase activity. Using an unbiased scRNA-seq approach, we systematically characterized the immunological impacts and signaling events upon overexpression of ENPP1’s catalytic activity in orthotopically implanted 4T1 cancer cells. We found that ENPP1-high cancer cells promote breast tumor growth by shunting the immunostimulatory cGAMP–STING pathway to the immunosuppressive eADO pathway, while fostering an angiogenic TME for tumor survival (*SI Appendix*, Fig. S5*D*). Using the *Enpp1^H362A^* variant that specifically abolishes ENPP1’s cGAMP hydrolysis activity and orthogonal molecular sponges to deplete extracellular cGAMP, we confirmed that cGAMP is the relevant substrate in in vivo cancer models in a manner dependent on downstream STING signaling. Together, our results demonstrate the importance of extracellular cGAMP–STING activation in antitumoral immunity and ascribe ENPP1 as an innate immune checkpoint of the extracellular cGAMP–STING pathway.

ENPP1’s contribution to different stages of tumor development including initiation, progression, and metastasis was not well understood. Importantly, our work provides evidence that ENPP1 promotes breast cancer initiation (Fig. 5*B*). Comparing between primary tumors and metastases, we noticed a stronger contribution of cGAMP–STING inhibition to the pro-metastatic phenotype of ENPP1 in our scRNA-seq analyses. We posit that this could be due to elevated cGAMP production along the oncogenic trajectory, as we showed that CIN-high pro-metastatic *Kif2c*+ cancer cells (17) expressed higher levels of *Cgas* (Fig. 3*A*). While a previous study attributed the increasing role of cGAMP hydrolysis by ENPP1 in metastasis to it replacing ATP as the major source of eADO (30), we raise an alternative explanation that direct dampening of cGAMP–STING activation is the culprit in metastasis. The causal link between ENPP1 levels in the TME and metastasis was established with the evidence that destroying ENPP1’s cGAMP hydrolysis activity phenocopied *Enpp1* deletion in completely abolished metastasis.

Prior studies supported a role for ENPP1 in promoting various cancer types but largely focused on ENPP1 expressed on the cancer cells (30, 48–51). Our scRNA-seq analyses revealed that not only is ENPP1 expressed on many host responder cells, but that responder-cell-derived ENPP1 has an outsized effect on blocking paracrine STING activation in those same cells. The importance of cancer- and responder-cell-derived ENPP1 in tumor development is in line with our understanding of paracrine extracellular cGAMP signaling as being short-ranged. ENPP1 on the surface of cGAMP-producing and cGAMP-sensing cells would be ideally poised to snatch a freshly exported or soon-to-be imported cGAMP molecule in close proximity to cGAMP transporters (26, 27, 29), thereby circumventing paracrine activation of the STING pathway within the TME. As cGAS is rarely inactivated in cancer cells (17) and there is no known intracellular cGAMP hydrolase (30), we bring forward a model that cancer cells export the high levels of cGAMP they produce, capitalizing on both cancer-cell- and responder-cell-derived ENPP1 for its extracellular clearance, to achieve immune evasion.

While this study focused on the roles of ENPP1 in the context of breast cancers, our findings could potentially be generalized to other types of immunologically cold tumors. We hypothesize that ENPP1 also plays immunosuppressive roles in tumor types where either the cancer cells or the TME highly expresses ENPP1. We showed that ENPP1 is broadly expressed by immunosuppressive immune cells such as M2-like macrophages and stromal cells such as myCAFs. In addition, we observed increased *Enpp1* expression in myCAFs in both primary and metastatic niches of ENPP1^T238A-OE^ 4T1 compared to ENPP1^WT-OE^ 4T1, suggesting ENPP1 expression is inducible either directly or indirectly by extracellular cGAMP and/or ATP. We therefore hypothesize ENPP1 inhibition should be investigated for tumors with high myeloid content and fibrosis. Future examination of ENPP1 in other mouse cancer models and patient cohorts are warranted to test these hypotheses. Future mechanistic studies on soluble factors, signaling pathways, and transcription factors that induce ENPP1 expression in the TME could lead to additional diagnostic and therapeutic insights.

Together, our detailed understanding of the molecular and cellular mechanisms of ENPP1 and the paracrine cGAMP–STING pathway shed light on clinical translations. For example, our data highlight the notion that host-derived ENPP1 is not a passive bystander, but rather actively involved in shaping the tumor immune microenvironment. We hypothesize that the ENPP1 status of the tissue in which cancer develops, either as the primary site or the site of metastasis, together with ENPP1 allele or expression level variations, could dictate the extent of ENPP1’s role in tumor development and even the risk of tumor development altogether. Furthermore, the ISPY 2 Trial data nominated ENPP1 as a potential companion diagnostic biomarker: *ENPP1*-low breast cancer patients are significantly more likely benefit from anti-PD-1 and PARPi therapies than their ENPP1-high counterparts. Conversely, we predict that *ENPP1*-high breast cancer patients will greatly benefit from a combination of ENPP1 inhibition with anti-PD-1/anti-PD-L1 or PARPi treatments. As a central player dictating cancer-innate-adaptive immune communication through the STING pathway, ENPP1 is a promising target for cancer immunotherapy that may bolster our arsenal of ICB therapeutics as a druggable innate immune checkpoint.

## Methods

See *SI Appendix* *f*or detailed methods.

### Mouse Strains.

C57BL/6J (strain #000664), BALB/cJ (strain #000651), C57BL/6J-*Sting^gt^/J* (strain #017537), C57BL/6J-*Enpp1^asj/GrsrJ^* (strain #012810), BALB/cJ-*Enpp1^asj-2J/GrsrJ^* (strain #019107), and FVB/N-Tg(MMTV-PyVT) 634Mul/J (strain #002374) mice were purchased from the Jackson Laboratory. C57BL/6J-*Enpp1^H362A^* and C57BL/6J-*Enpp1^H362A^* × *Sting1^−/−^*mice were generated and characterized in house (Carozza et al., 42). FVB/N-Tg(MMTV-PyVT) 634Mul/J mice were bred with C57BL/6J-WT or C57BL/6J-*Enpp1^H362A^* to generate B6;FVB-MMTV and B6;FVB-*Enpp1^H362A^* × MMTV respectively. For MMTV spontaneous tumor model, female mice from the second generation of both B6;FVB-MMTV and B6;FVB-*Enpp1^H362A^* × MMTV genotypes were used for experiment. For all other breast tumor experiments, female mice between 6 and 15 wk old were used for tumor experiments. Mice were maintained at Stanford University in compliance with the Stanford University Institutional Animal Care and Use Committee regulations. All procedures were approved by the Stanford University Administrative Panel on Laboratory Animal Care (APLAC).

### [^32^P] cGAMP Degradation Thin-Layer Chromatography Assays.

For experiments reported in *SI Appendix*, Fig. S1 *A*–*C*, the cGAMP degradation activity assay (20 μL) for cells containing 50% cell lysate, cGAMP (1 μM, with trace [^32^P] cGAMP spiked in), and standard ENPP1 activity buffer (50 mM Tris pH 9, 250 mM NaCl, 0.5 mM CaCl_2_, 1 μM ZnCl_2_) took place in room temperature. For experiments reported in *SI Appendix*, Fig. S6 *A* and *B*, the cGAMP degradation activity assay for mouse organs (30 μL) containing 75% organ lysate (100 mg/mL), 5 μM cGAMP, and PBS took place in 37 °C. Last, cGAMP degradation activity assay (20 μL) for mouse serum containing 50% serum, 5 μM cGAMP and physiological ENPP1 activity buffer (50 mM Tris pH 7.5, 150 mM NaCl, 1.5 mM CaCl_2_, and 10 μM ZnCl_2_) took place in 37 °C. At indicated times, 1 μL aliquots of the reaction were quenched by spotting on HP-TLC silica gel plates (Millipore). The TLC plates were run in mobile phase (85% ethanol, 5 mM NH_4_HCO_3_) and exposed to a phosphor screen (GE BAS-IP MS). Screens were imaged on a Typhoon 9400 scanner and the ^32^P signal was quantified using ImageJ. The sample size and statistical tests of computation are indicated in the respective figure legend.

### 4T1 Murine Breast Tumor Models.

For experiment reported in Fig. 1, BALB/cJ female mice were orthotopically injected with 2.5 × 10^6^ ENPP1^WT-OE^ or ENPP1^T238A-OE^ 4T1 suspended in 100 μL of PBS cells in the 4th mammary fat pad (MFP). When tumors reached 1,000 mm^3^, we killed the animals and collected primary tumors and lungs. Tissues were processed into single-cell suspension following steps described above. Half of the lung suspension were plated into 60 μM 6-thioguanine (Sigma-Aldrich) and 10% heat-inactivated (fetal bovine serum) FBS (R&D Systems) containing Iscove’s Modified Dulbecco’s Medium (ThermoFisher) media and cultured for 6 to 12 d without disturbance. At the end of the experiment, colonies of metastases were fixed in methanol and visualized with 0.03% (w/v) methylene blue (Sigma-Aldrich). Metastatic colonies were quantified with Fuji Image J. The rest half of the lung suspension and the tumor suspension were cryopreserved and later thawed for scRNA-seq. For primary tumor experiment reported in Fig. 4*A* and *SI Appendix*, Fig. S6 *A* and *B*, we orthotopically injected 5 × 10^4^ WT or *Enpp1^−/−^* 4T1-luc cells into WT or *Enpp1^−/−^* BALB/cJ. When tumors were palpable with an average tumor volume of 100 ± 20 mm^3^ (determined by length^2^ × width / 2), 10 to 12 d after cell inoculation, tumors were irradiated with 12 Gy using a 225-kVp cabinet X-ray irradiator filtered with 0.5-mm Cu (IC-250, Kimtron Inc., CT) following previously described procedures (Carozza, Böhnert et al., 23). We killed mice when their tumors reached 1,000 mm^3^ and collected their primary tumors and sera for cGAMP degradation activity following steps described above. For tumor metastasis experiment reported in Fig. 4*B* and *SI Appendix*, Fig. S6*D*, we intravenously injected 5 × 10^4^ WT or *Enpp1*^−/−^ 4T1 cells into the tail veins of WT or *Enpp1^−/−^* BALB/cJ mice. We collected their lungs around day 30 and quantified metastaticburden following process describe above. For tumor metastasis experiment reported in *SI Appendix*, Fig. S6*C*, we orthotopically injected 2.5 × 10^4^ WT or *Enpp1^−/−^* 4T1 cells into the tail veins of WT or *Enpp1^−/−^* BALB/cJ mice, respectively. On day 33, we killed the animals and collected blood, dLNs, primary tumors, lungs, livers, and brains for metastasis assay. The sample size and statistical tests of computation are indicated in the respective figure legend.

### E0771 Murine Breast Tumor Models.

For primary tumor experiment in Fig. 5*A*, we orthotopically injected 2.5 × 10^4^ E0771 cells into the 4th MFP of WT, *Enpp1^H362A^*, *Sting1^−/−^,* or *Enpp1^H362A^* × *Sting1^−/−^* C57BL/6J mice. We measured animal survival by the time it took for the tumors to reach 1,000 mm^3^. For tumor metastasis experiment in Fig. 5*C* and *SI Appendix*, Fig. S6*E*, we intravenously injected E0771.lmb.PuroR cells into the tail veins of WT, *Enpp1^H362A^*, *Enpp1^−/−^*,*Sting1^−/−^,* and *Enpp1^H362A^* × *Sting1^−/−^* C57BL/6J mice. Then, 30 d later, we cultured dissociated lungs in 1 μg/μL puromycin for 9 d before methylene blue visualization. The sample size and statistical tests of computation are indicated in the respective figure legend.

### MMTV-PyMT Spontaneous Breast Tumor Model.

Female mice hemizygous for the MMTV-PyMT transgene and homozygous for the wild type or H362A mutated *Enpp1* gene from the second generation were included in experiments. Individual tumor volume (determined by 0.5 × length × width^2^) was added up to yield total tumor volume. Tumor onset was defined by the date on which a palpable tumor of more than 0.5 mm^3^ was observed. Tumor-free survival was plotted, and statistical significance was assessed by the log-rank Mantel-Cox test.

### FACS Analysis of Tumors upon *Enpp1* Knockout and/or cGAMP Depletion.

5 × 10^4^ WT or *Enpp1^−/−^* 4T1-luc cells were orthotopically injected into WT or *Enpp1^−/−^* BALB/cJ mice respectively. Starting the next day, mice were intratumorally injected with 100 μL of 100 μM neutralizing (WT) or non-binding (R237A) STING every other day up to day 13. Mice were killed on day 15 and tumors were collected and digested in Roswell Park Memorial Institute (PRMI) + 10% FBS with 20 μg/ml DNase I type IV (Sigma-Aldrich) and 1 mg/mL Collagenase from Clostridium histolyticum (Sigma-Aldrich) at 37 °C for 30 min. Tumors were passed through a 100 μm cell strainer (Fisher Scientific) and red blood cells were lysed using red blood cell lysis buffer (155 mM NH_4_Cl, 12 mM NaHCO_3_, and 0.1 mM Ethylenediaminetetraacetic acid) for 5 min at room temperature. Cells were stained with Live/Dead fixable near-IR or blue dead cell staining kit (ThermoFisher). Samples were then fixed and permeabilized with eBioscience FOXP3/Transcription Factor Staining Buffer Set (Invitrogen), Fc-blocked for 10 min using TruStain fcX (BioLegend) and subsequently antibody-stained with antibodies. Cells were analyzed using a Symphony (BD Biosciences), or an Aurora analyzer (Cytek). Data was analyzed using FlowJo V10 software (BD) and Prism 9.1.0 software (GraphPad) for statistical analysis and statistical significance was assessed using the unpaired *t* test with Welch’s correction.

### scRNA-seq of Murine Primary Tumors and Lungs with Metastases.

MFP primary tumors and lungs containing metastases were harvested from two ENPP1^WT-OE^ or ENPP1^T238A-OE^ tumor–bearing mice when primary tumors reached 1,000 mm^3^ (Fig. 1). Cryopreserved single-cell suspensions were thawed into warm RPMI media with 10% HI-FBS. Cells were washed with PBS and 0.04% w/v BSA, passed through a 40-μm Flowmi cell strainer (Bel-Art, 974-25244), and assessed for concentration and viability with an automated cell counter. Cells were resuspended in PBS and 0.04% w/v BSA to 1,000 cells/μL. Single-cell suspensions were processed with a Chromium Controller microfluidic device (10× Genomics), using the Chromium Next GEM Single-Cell 3’ HT Reagent Kits v3.1 (Dual Index). Library preparation was performed according to the manufacturer's instructions (single-cell 3’ HT reagent kits v3.1 protocol, Rev D, 10× Genomics). Briefly, cells were incorporated into gel bead-in-emulsions (GEM) and reverse-transcribed. The pooled barcoded cDNA was then cleaned up with Silane DynaBeads, amplified by PCR and the appropriate molecular weight fragments were selected with SPRIselect reagent for subsequent library construction. During the library construction Ilumina, R2 primer sequence, paired-end constructs with P5 and P7 sequences and a sample index were added. Pooled libraries were sequenced on NextSeq 2000 (Illumina). FASTQs were demultiplexed, mapped to the murine reference genome (Ensembl release 93 GRCm38), and gene counts were quantified using Cell Ranger (version 3.1.0). In total, we obtained 653,549,810 reads across 38,637 cells, and detected on average (median) 1,445 genes per cell.

### scRNA-seq Clustering and Cell Type Annotation.

All subsequent processing, quality control, and analyses were performed with Cellenics (https://scp.biomage.net/data-management). We filtered out cells with high mitochondrial content and high doublet count. We then applied Harmony data integration with HVGs = 2,000 and performed dimensionality reduction using 27 principal components (90.18% variation). We performed Louvain clustering with resolution set to be 0.8. We manually annotated 18 clusters based on known cell markers (*SI Appendix*, Fig. S2*A*). Specifically, we identified cancer cells based on markers enriched in 4T1 compared to BALB/cJ MFP (52). A separate cancer cluster was annotated for overexpressing *Kif2c*, which has been reported to drive CIN and metastasis (17) and additional genes related to metastasis (52). Among fibroblasts, a unique cluster enriched in tumors overexpresses myofibroblast marker *Acta2* as well as *Tgfb1* and *Tgfb2* and are known as myCAF and overtly immunosuppressive (41, 53). We also performed detailed subcluster analysis on macrophages (*SI Appendix*, Fig. S3 *A* and *B*) and T cells (*SI Appendix*, Fig. S3 *C* and *D*). Cluster frequency analysis, gene expression analysis, and differential gene expression are performed in Cellenics.

### The ISPY 2 Trial Patient Dataset Analysis.

Normalized, batch-corrected de-identified microarray data and sample metadata were retrieved from GSE194040. Individual two-tailed *t* tests were performed comparing ENPP1 expression between patients who achieved a pCR and those who did not, within each ISPY 2 Trial clinical arm. Similarly, ENPP1 expression was compared between tumors that were likely to respond to immunotherapy (immune+) and those that were not using two-tailed *t* tests. Immune+ status was estimated based on the average dendritic cell and STAT1 signatures (Wolf et al., 44).

## Supplementary Material

Appendix 01 (PDF)

## Data Availability

scRNA-seq raw and processed data can be accessed via GSE233659 (54). All data reported in this paper will be shared by the lead contact upon request. Any additional information required to reanalyze the data reported in this paper is available from the lead contact upon request.
