# Supplementary material for: ENPP1 is an innate immune checkpoint of the anticancer cGAMP–STING pathway in breast cancer
Source: Proc Natl Acad Sci U S A. 2023 Dec 20;120(52):e2313693120. doi: 10.1073/pnas.2313693120 (PMC10756298; doi:10.1073/pnas.2313693120)
Supplement: Supplementary file 1 — Appendix 01 (PDF) [file pnas.2313693120.sapp.pdf]

Supporting Information for

ENPP1 is an innate immune checkpoint of the anticancer cGAMP-STING pathway in breast cancer

Songnan Wang,[1,2,3] Volker Böhnert,[1,2] Alby J. Joseph,[1,2,3] Valentino Sudaryo,[1,2,3] Gemini Skariah,[1,2] Jason T. Swinderman,[3,4,5] Feiqiao B.Yu,[3] Vishvak Subramanyam,[4,5,6,7] Denise M. Wolf,[8] Xuchao Lyu,[2,9] Luke A. Gilbert,[3,4,5] Laura J. van't Veer, [8] Hani Goodarzi,[4,5,6,7] Lingyin Li[1,2,3*]

[1]Department of Biochemistry, Stanford University, Stanford, CA 94305, USA
[2]ChEM-H Institute, Stanford University, Stanford, CA94305, USA
[3]Arc Institute, Palo Alto, CA 94304, USA
[4]Department of Urology, University of California, San Francisco, San Francisco, CA 94143, USA
Helen Diller Family Comprehensive Cancer Center, University of California, San Francisco, San Francisco, CA 94158, USA.
[6]Department of Biophysics & Biochemistry, University of California, San Francisco, San Francisco, CA 94143, USA
Baker Computational Health Science Institute, University of California, San Francisco, CA 94143, USA
[8]Department of Laboratory Medicine, University of California, San Francisco, San Francisco, CA 94115, USA
[9]Department of Pathology, Stanford University School of Medicine, Stanford, CA 94305, USA

*Corresponding author: Lingyin Li. **Email:** lingyinl@stanford.edu

Table of Contents:

Figure S1. Generation of ENPP1$^{WT\text{-}OE}$ and ENPP1$^{T238A\text{-}OE}$ 4T1s and optimization of *ex vivo* metastasis culture.
Figure S2. scRNA-seq analysis of murine 4T1 primary tumors and lung metastases.
Figure S3. Subclustering of macrophages and T cells.
Figure S4. Expression of genes in the STING pathway.
Figure S5. Contribution of eADO pathway and HP secretion in ENPP1 overexpression.
Figure S6. Enpp1 knockout in cancer and tissue cells.

Appendix S1. Detailed Methods
1. Synthesis and purification of cGAMP and [^{32}P] cGAMP
2. STING expression and purification
3. Mammalian cell lines and primary cells
4. Recombinant DNA
5. Generation of transiently edited cell lines
6. Generation of stable expression cell lines
7. Serum and lysate preparation
8. Western blotting
9. Quantification and statistical analysis

Table S1. Reagent or Resources
Table S2. Oligonucleotide Sequences

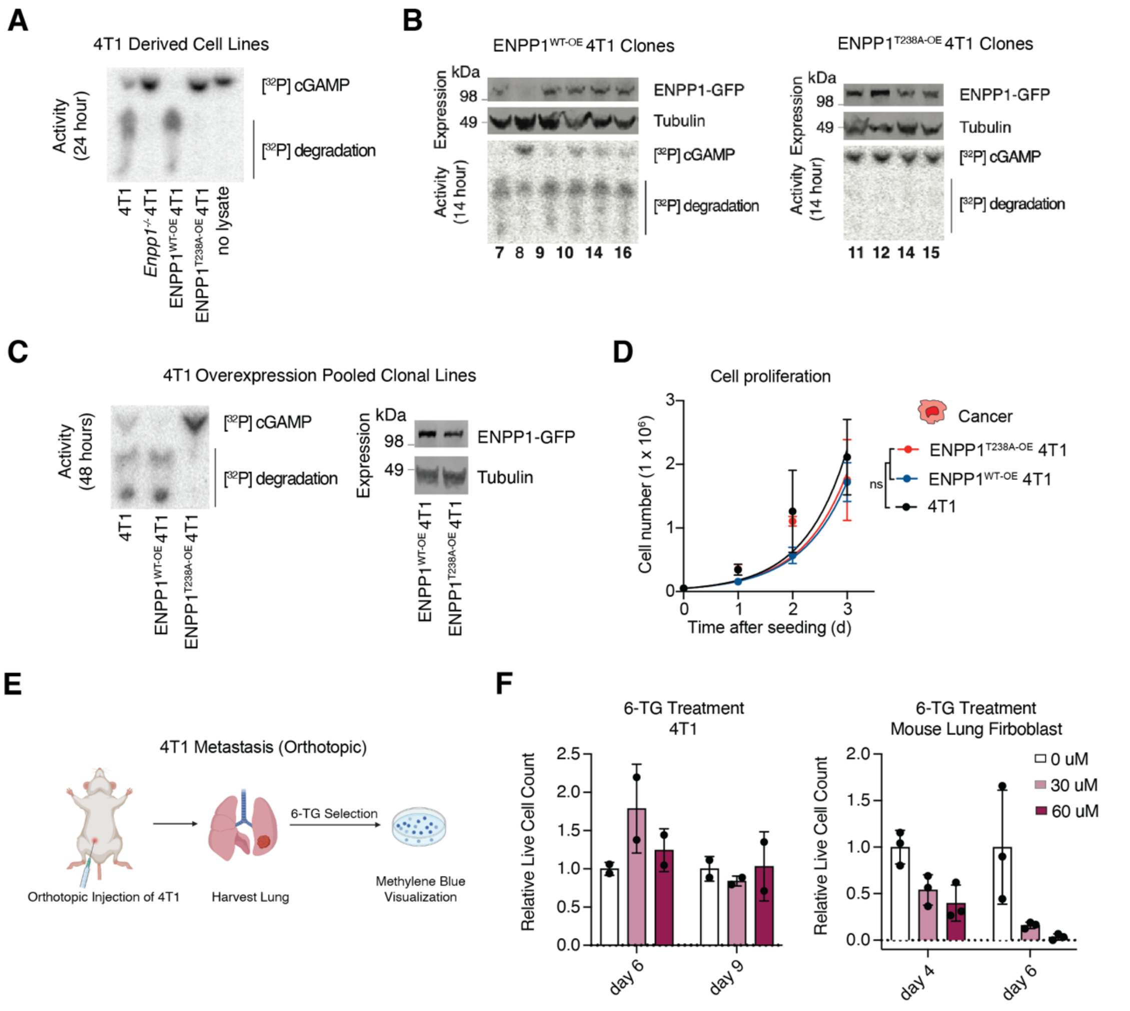

Figure S1. Generation of ENPP1$^{WT\text{-}OE}$ and ENPP1$^{T239A\text{-}OE}$ 4T1s and optimization of *ex vivo* metastasis culture

(A) ENPP1 degradation activity in 4T1 and its derived cell lines as assessed by TLC.

(B) ENPP1-GFP expression (top) and ENPP1 degradation activity (bottom) of ENPP1$^{WT\text{-}OE}$ 4T1 and ENPP1$^{T238A\text{-}OE}$ 4T1 clones as assessed by western blotting and TLC. Bolded clones were pooled for experiments.

(C) ENPP1-GFP degradation activity (left) and ENPP1 expression (right) of ENPP1$^{WT\text{-}OE}$ 4T1 and ENPP1$^{T238A\text{-}OE}$ 4T1 pooled clones as assessed by TLC and western blotting.

(D) Proliferation of ENPP1$^{WT\text{-}OE}$ and ENPP1T$^{238A\text{-}OE}$ 4T1 pooled clonal cell lines compared with WT 4T1 cells over time (n = 3 biological replicates).

(E) Experimental schematic of *ex vivo* culture of 4T1 metastasis by orthotopic injection.

(F) Number of live 4T1 cells (left) or mouse lung fibroblasts (right) 6 and 9 days after 0, 30, or 60 μM 6-thioguanine (6-TG) treatment.

Data were plotted as mean $\pm$ SD. *P* values were determined by unpaired *t* test.

TLC stands for thin layer chromatography.

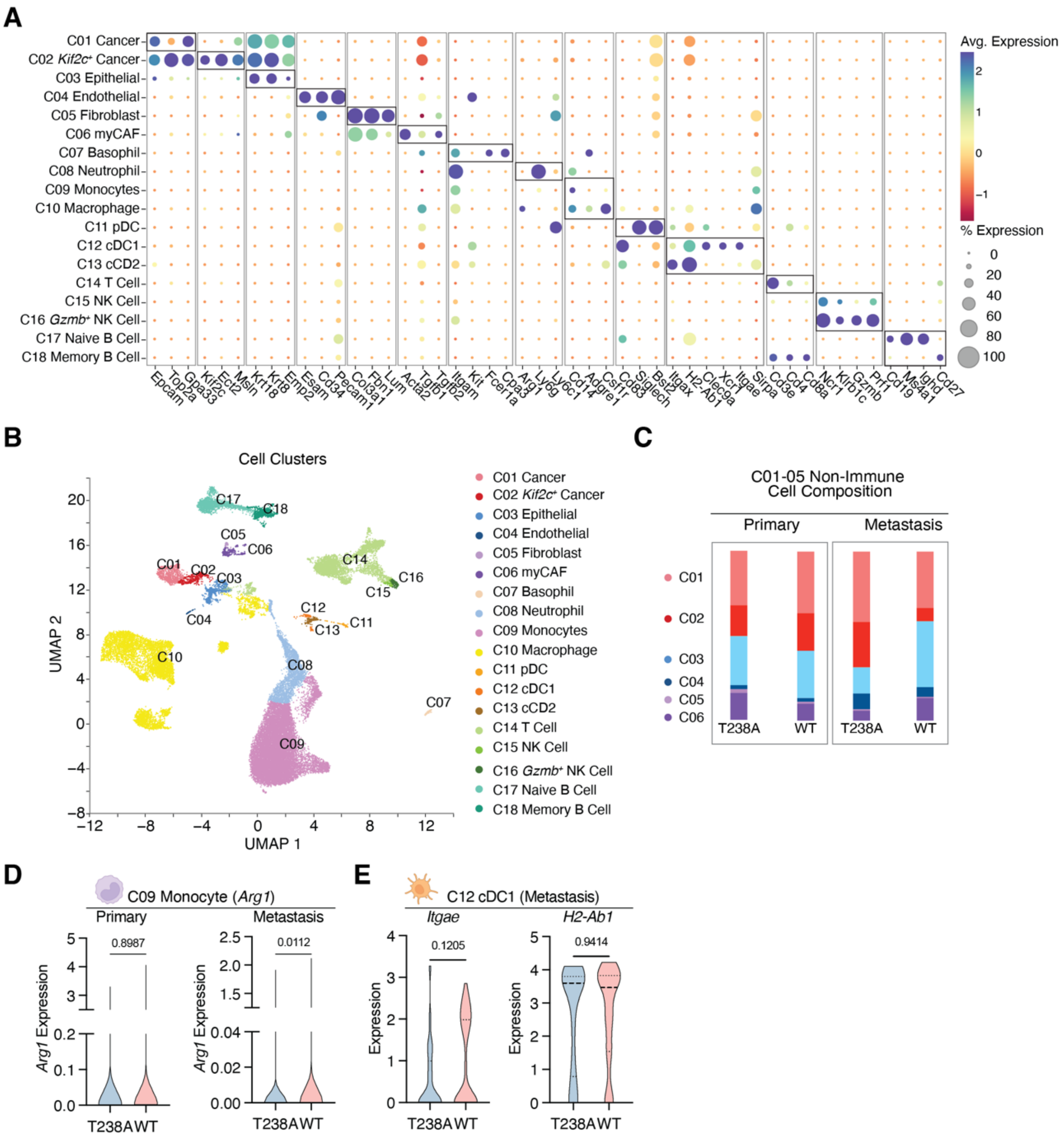

Figure S2. ScRNA-seq analysis of 4T1 murine primary tumors and lung metastases
(A) Bubble heatmap showing expression of selected marker genes for cluster annotation. Dot size indicates fraction of expressing cells, colored based on average expression levels.
(B) UMAP plot of the annotated clusters of ENPP1$^{T238A-OE}$ and ENPP1^{WT-OE} 4T1 primary tumors and metastasis colonized lungs.
(C) Barplots comparing non-immune cell compositions (containing C01-C05) between ENPP1$^{T238A-OE}$ and ENPP1^{WT-OE} 4T1 primary tumors and metastasis colonized lungs.
(D and E) Violin plots of indicated transcripts of indicated cell types comparing between ENPP1$^{T238A-OE}$ and ENPP1^{WT-OE} 4T1 tumors or metastases. *Arg1* in monocytes in primary tumors and lung metastases (D); *Itgae* and *H2-Ab1* in cDC1s in lung metastases (E). *P* values were determined by nonparametric Mann-Whitney *U* test.
cDC1 stands for conventional dendritic cell type 1.

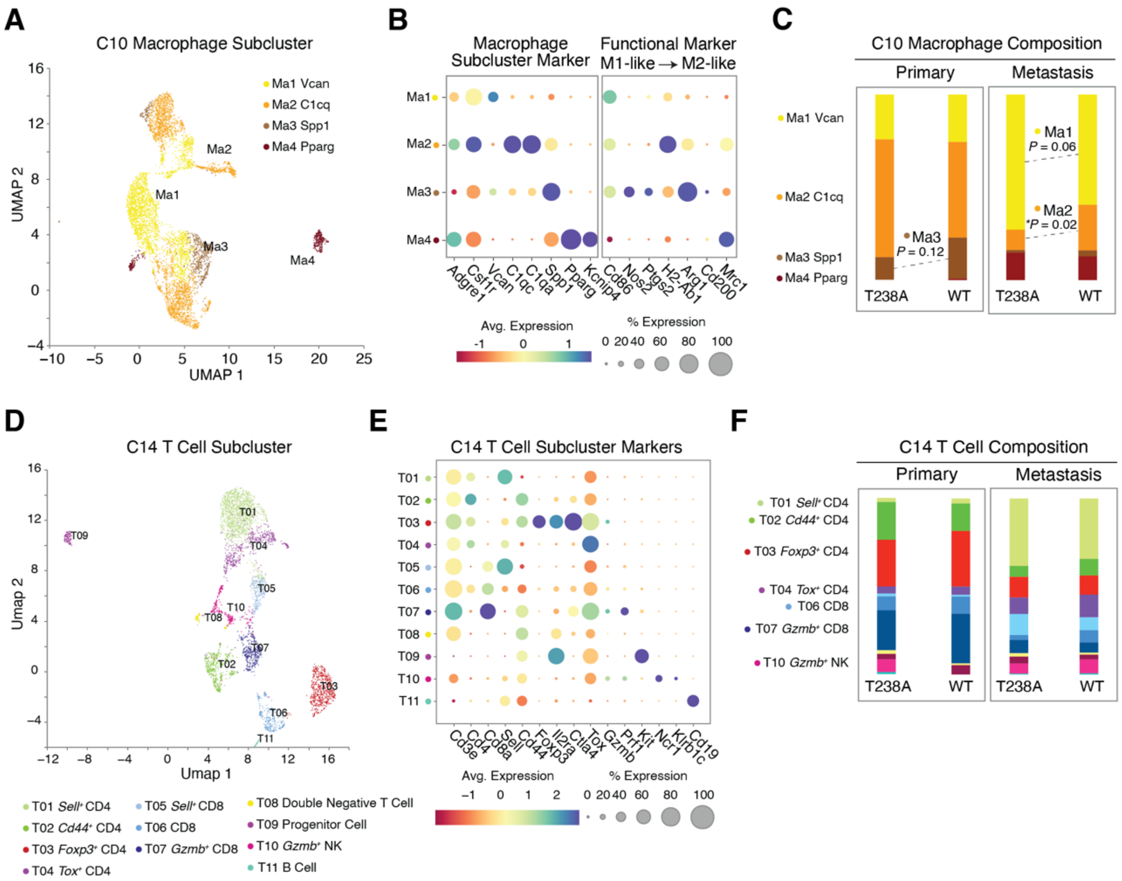

Figure S3. Subclustering of macrophages and T cells

(A) UMAP plot of the annotated subclusters of macrophages.

(B) Bubble heatmap showing expression of marker genes for macrophage subcluster annotation and functional markers indicating M1-like versus M2-like macrophage cell phenotypes. Dot size indicates fraction of expressing cells, colored based on average expression levels.

(C) Barplots comparing macrophage subcluster compositions (containing Ma1-Ma4) between ENPP1$^{T238A\text{-}OE}$ and ENPP1$^{WT\text{-}OE}$ 4T1 primary tumors and metastasis colonized lungs.

(D) UMAP plot of the annotated subclusters of T cells.

(E) Bubble heatmap showing expression of marker genes for T cell subcluster annotation. Dot size indicates fraction of expressing cells, colored based on average expression levels.

(F) Barplots comparing T cell subcluster compositions (containing T01-T11) between ENPP1$^{T238A\text{-}OE}$ and ENPP1$^{WT\text{-}OE}$ 4T1 primary tumors and metastasis colonized lungs.

P values were determined by unpaired *t* test. $^*P \leq 0.05$.; *P* value is shown if it is between 0.05 - 0.15.

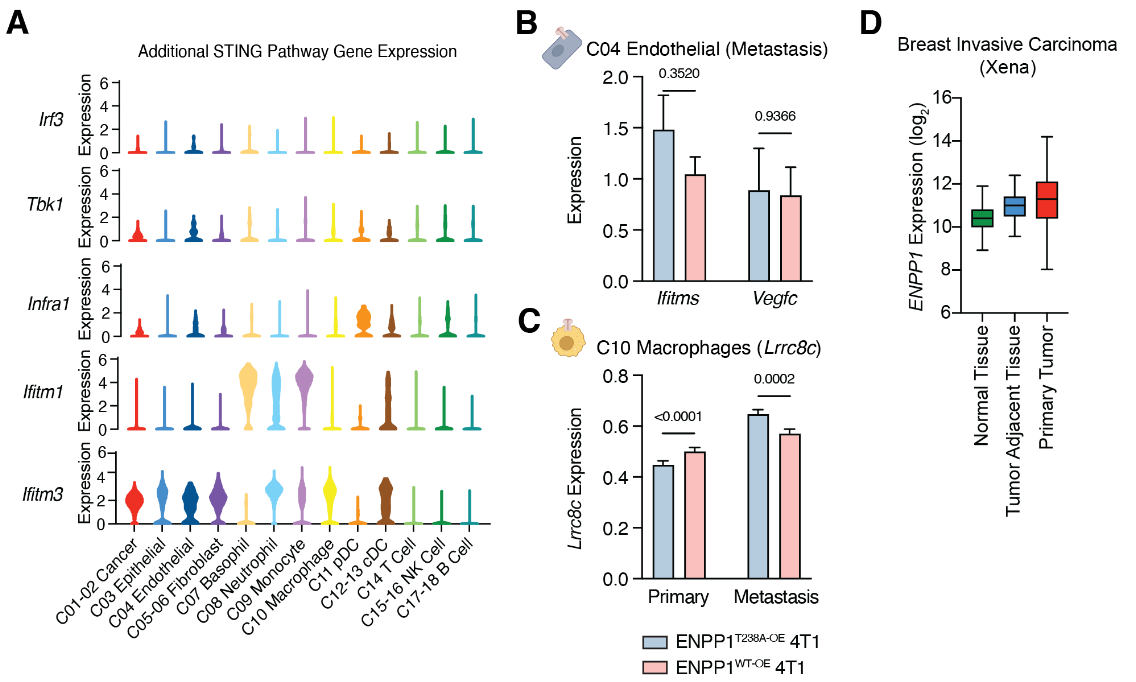

Figure S4. Expression of additional genes in the STING pathway

(A) Violin plots of *Irf3*, *Tbk1*, *Infra1*, *Ifitm1*, and *Ifitm3* across the annotated clusters.

(B-C) Bar graphs of indicated transcripts in indicated cell types comparing between ENPP1$^{T238A\text{-}OE}$ and ENPP1$^{WT\text{-}OE}$ 4T1 tumors or metastases. *Ifitms* and *Vegfc* in endothelial cells (C04) in lung metastases (B); *Lrrc8c* in macrophages (C10) in primary tumors and lung metastases. Shown as mean ± SEM. *P* values were determined by nonparametric Mann-Whitney *U* test.

(D) Bar graphs of *ENPP1* expression in normal breast tissue (*n* = 179), tumor adjacent tissue (*n* = 113), and primary tumor (*n* = 1092) in breast invasive carcinoma. Shown as box plots of median and interquartile levels. RNA sequencing data for normal tissue are from GTEx database, and for tumor adjacent tissue and primary tumor are from TCGA database, all queried using UCSC Xena portal.

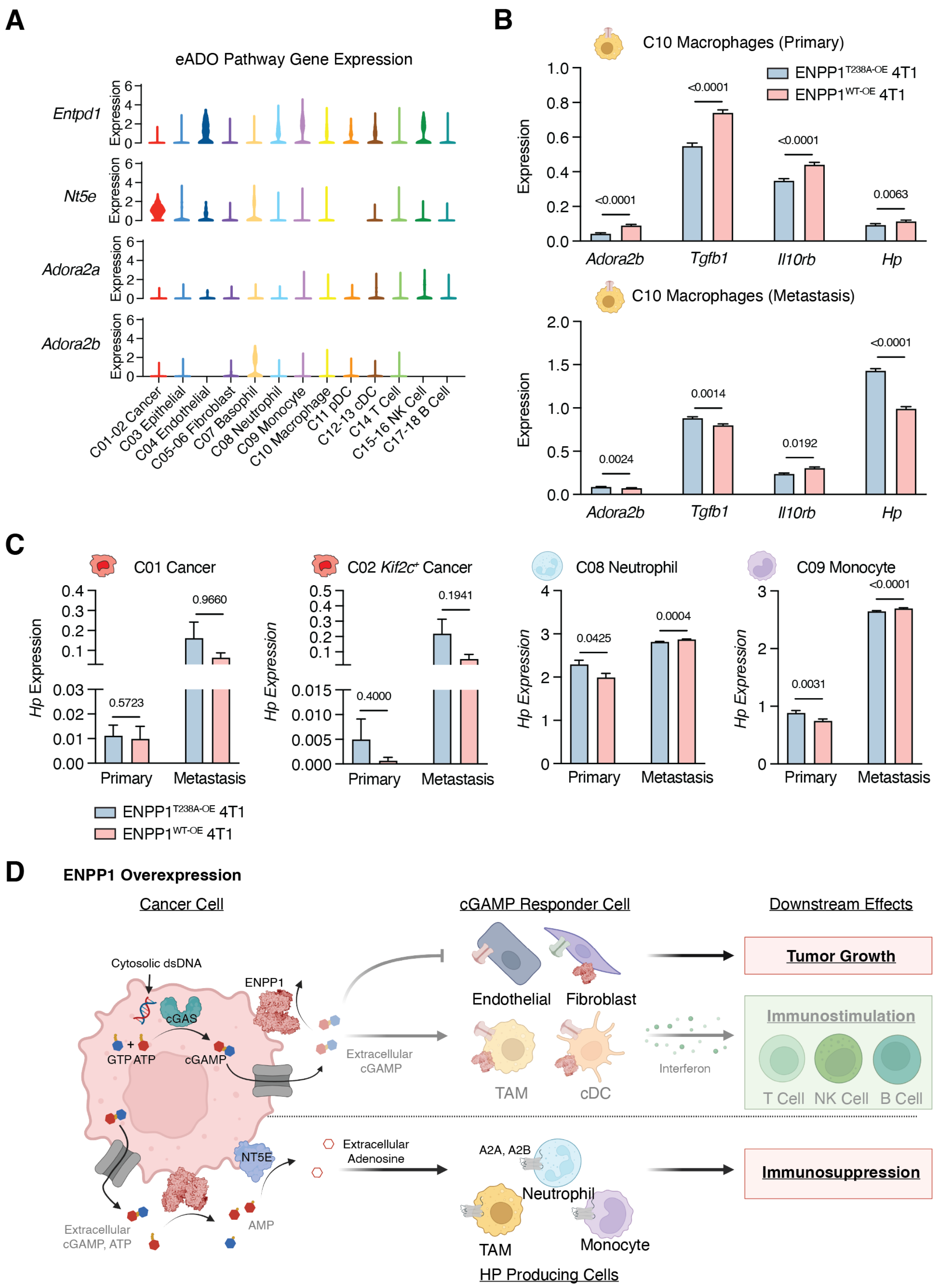

Figure S5. Contribution of eADO pathway and HP secretion in ENPP1 overexpression
(A) Violin plots of *Entpd1*, *Nt5e*, *Adora2a* and *Adora2b* across the annotated clusters.
(B-C) Bar graphs of indicated transcripts in indicated cell types comparing between ENPP1$^{T238A-OE}$ and ENPP1^{WT-OE} 4T1 tumors or metastases. *Adora2b*, *Tgfb1*, *Il10rb*, *Hp* in macrophages in

primary tumors and lung metastases (B); *Hp* in cancer cells, *Kif2c*$^+$ cancer cells, neutrophils, and monocytes in primary tumors and lung metastases (C).
(D) Proposed model of mechanism of action in ENPP1 overexpression.
Bars represent mean $\pm$ SEM. *P* values were determined by nonparametric Mann-Whitney *U* test. HP stands for Haptoglobin. TAM stands for tumor-associated macrophages. cDC stands for conventional dendritic cell.

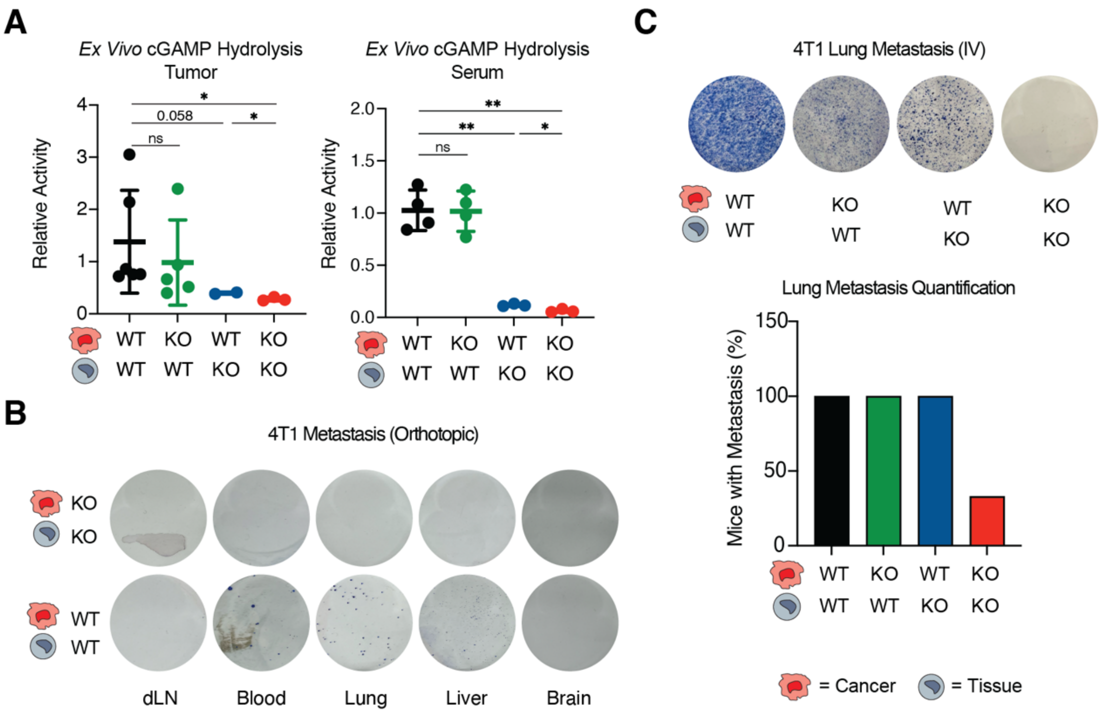

Figure S6. *Enpp1* knockout in cancer and tissue cells

(A) Relative cGAMP hydrolysis activity calculated from kinetic analysis by tumor lysates and sera from randomly selected mice in Figure 4A reaching experimental endpoint (n = 2-6 biological replicates). Data represent mean $\pm$ SD. *P* values were determined by unpaired *t* test with Welch correction.

(B) Images of metastatic colonies of WT or *Enpp1*$^{-/-}$ 4T1 BALB/cJ orthotopically injected with WT or *Enpp1*$^{-/-}$ 4T1 respectively.

(C) Representative images of lung metastatic colonies and the percentage of mice with lung metastasis from Figure 4B. WT or *Enpp1*$^{-/-}$ 4T1 BALB/cJ mice were intravenously injected with WT or *Enpp1*$^{-/-}$ 4T1 (n = 4, 5, 3, 3 mice for *Enpp1* KO x KO, KO x WT, WT x KO, and WT x WT cancer x tissue genotype combinations).

Appendix S1. Detailed Methods

1. Synthesis and purification of cGAMP and [^{32}P] cGAMP
To enzymatically synthesize cGAMP (Ritchie *et al.*, 2019), 1 μM purified sscGAS was incubated testis DNA (Sigma) for 24 h. The reaction was then heated at 95°C for 3 min and filtered through a 3-kDa filter. cGAMP was purified from the reaction mixture using a PLRP-S polymeric reversed phase preparatory column (100 Å, 8 μm, 300 x 25 mm; Agilent Technologies) on a preparatory HPLC (1260 Infinity LC system; Agilent Technologies) connected to UV-vis detector (ProStar; Agilent Technologies) and fraction collector (440-LC; Agilent Technologies). The flow rate was set to 25 mL/min. The mobile phase consisted of 10 mM triethylammonium acetate in water and acetonitrile. The mobile phase started as 2% acetonitrile for the first 5 min. Acetonitrile was then ramped up to 30% from 5-20 min, then to 90% from 20-22 min, maintained at 90% from 22-25 min, and then ramped down to 2% from 25-28 min. Fractions containing cGAMP were lyophilized and resuspended in water. The concentration was determined by measuring absorbance at 280 nm. To enzymatically synthesize [^{32}P] cGAMP, 1 μM purified sscGAS was incubated with 20 mM Tris-HCl pH 7.4, 250 μCi (3000 Ci/mmol) [α-^{32}P] ATP (Perkin Elmer), 1 mM GTP, 20 mM $MgCl_2$, and 100 μg/mL herring testis DNA (Sigma) in a reaction volume of 100 μL for 24 h. The reaction was purified by preparatory TLC on a HP-TLC silica gel plate (Millipore), eluted in water, and filtered through a 3-kDa filter to remove silica gel.

2. STING expression and purification
WT neutralizing STING or R237A non-binding STING were expressed and purified using previously published methods (Carozza, Böhnert, *et al.*, 2020). In brief, pTB146 His-SUMO-mSTING (residues 139-378) was expressed in Rosetta (DE3) pLysS competent cells (Sigma-Aldrich). Cells were grown in 2xYT medium with 100 μg/mL ampicillin until they reached an OD600 of 1. They were then induced with 0.75 mM IPTG at 16°C overnight. Cells were pelleted and resuspended in 50 mM Tris pH 7.5, 400 mM NaCl, 10 mM imidazole, 2 mM DTT, and cOmplete protease inhibitors (Sigma-Aldrich). The cells were then flash frozen and thawed twice before sonication to lyse the cells. The supernatant was incubated with HisPur cobalt resin (Thermo Scientific) for 30 min at 4°C. The resin-bound protein was washed with 50 column volumes of 50 mM Tris pH 7.5, 150 mM NaCl, 2% triton X-114; 50 column volumes of 50 mM Tris pH 7.5, 1 M NaCl; and 20 column volumes of 50 mM Tris pH 7.5, 150 mM NaCl. Protein was eluted from resin with 600 mM imidazole in 50 mM Tris pH 7.5, 159 mM NaCl. Fractions containing His-SUMO-STING were pooled, concentrated, and dialyzed against 50 mM Tris pH 7.5, 150 mM NaCl while incubating with SUMOlase His-ULP1 to remove the His-SUMO tag overnight. The solution was incubated with the HisPur cobalt resin again to remove the His-SUMO tag, and STING was collected from the flowthrough. Protein was dialyzed against 20 mM Tris pH 7.5, loaded onto a HitrapQ anion exchange column (GE Healthcare) using Äkta FPLC (GE Healthcare), and eluted with a NaCl gradient. Fractions containing STING were pooled, buffer exchanged into PBS, and stored at -80°C until use.

3. Mammalian cell lines and primary cells
4T1 and E0771.lmb cells were procured from ATCC. E0771 cells were procured from CH3 BioSystems. 4T1-luciferase (4T1-luc) cells were a gift from C. Contag, Stanford University, Stanford, CA, USA (Vilalta *et al.*, 2014). 4T1-luc *Enpp1*$^{-/-}$ pooled clonal cell line and 293T cGAS *ENPP1*$^{-/-}$ single clonal cell line were generated in a previous study (Carozza, Böhnert, *et al.*, 2020). Primary mouse lung fibroblasts were isolated by incubating the minced lungs with 1 mg/mL collagenase from *Clostridium histolyticum* (Sigma-Aldrich) and 20 μg/mL DNase I (Sigma-Aldrich) for 1 hour, then passed through 100 μM cell strainer, spun down, treated with RBC lysis buffer for 5 minutes and plated in 10cm dish until all fibroblasts attached. To isolate metastasis from the blood or distant organs in animal studies, blood, draining inguinal lymph nodes (dLNs), lungs, livers, and brains were collected at experimental end point. Blood was dispensed into a 15-ml conical tube containing 10 mL 1x HBSS (Gibco), centrifuged at 1500 rpm at room temperature for 5 minutes before plated. dLNs were teased apart by forcing through a 100 μM strainer before plated. All other organs were minced with scissors and forces and digested in collagenase from

Clostridium histolyticum (Sigma-Aldrich) at various conditions: 4°C for 75 minutes for lungs, 37°C for 30 minutes for livers, and 37°C for 120 minutes for brains. Digested organs were passed through a 100 μM cell strainer, washed twice with 1x HBSS (Gibco) before plating. 4T1, E0771, E0771.lmb, and their derived cell lines were maintained in RPMI (Corning Cellgro) supplemented with 10% FBS (R&D Systems), 10 mM HEPES (Gibco), and 1% penicillin-streptomycin (ThermoFisher). 293T cGAS *ENPP1*$^{-/-}$ and mouse lung fibroblast cells were maintained in DMEM (Corning Cellgro) supplemented with 10% FBS and 1% penicillin-streptomycin. 4T1 and E0771.lmb.PuroR metastasis were cultured for 7-10 days with minimal disturbance in IMDM (Gibco) supplemented with 10% FBS, 1% penicillin-streptomycin, and 60 μM 6-thioguanine (Sigma-Aldrich) or 1.5 μg/mL puromycin (Sigma-Aldrich) respectively. All cells were maintained in a humidified incubator at 37°C and 5% CO_2. All cell lines tested negative for mycoplasma contamination.

4. Recombinant DNA

To clone guide RNA plasmid targeting *Enpp1*, *Enpp1* guide sequences listed in **Table S2** were cloned into the BbsI site of PX458-GFP or P458-mCherry backbones (synthesized by Addgene) following the protocol from Ryuji Morizane Lab at Harvard in order. To clone pLenti-CMV-mENPP1-WT-GFP-Puro plasmid, mENPP1-WT sequence was amplified from pcDNA3-mENPP1-FLAG (synthesized by Genscript) using pLenti_mENPP1_fwd and pLenti_mENPP1_rev primers in **Table S2** and inserted into the XbaI-BamHI sites of pLenti-CMV-GFP-Puro (Addgene). To clone pLenti-CMV-mENPP1-T238A-GFP-Puro plasmid, T238A point mutants were first introduced into pcDNA3-mENPP1-FLAG using QuikChange mutagenesis. mENPP1-T238A sequence was then introduced into the XbaI-BamHI sites of pLenti-CMV-GFP-Puro. pLenti-TetONE-FLAG-Puro were synthesized by Addgene and used to generate E0771.lmb.PuroR cell line. All oligonucleotide sequences are in **Table S2**.

5. Generation of transiently edited cell lines

4T1 *Enpp1*$^{-/-}$ cells were created the same way as 4T1-luc *Enpp1*$^{-/-}$ cells previously described (Carozza, Böhnert, *et al.*, 2020). Briefly, 4T1 underwent transient transfection with Lipofectamine 3000 of the following pairs of sgRNAs targeting mouse *Enpp1*: PX458-mENPP1_sgRNA1-GFP and PX458- mENPP1_sgRNA2-mCherry; PX458- mENPP1_sgRNA3-GFP and PX458-mENPP1_sgRNA1-mCherry. Double GFP and mCherry positive cells were FACS sorted and underwent single-cell cloning. Sequence knockout was confirmed with PCR using mENPP1_sgRNA12_seq_fwd and mENPP1_sgRNA12_seq_rev, or mENPP1_sgRNA34_seq_fwd and mENPP1_sgRNA34_seq_rev primer pairs. Functional knockout was confirmed with activity assay (commercial antibodies are not sensitive enough for verification of protein expression). Multiple clean knockout clones were pooled to generate the 4T1 *Enpp1*$^{-/-}$ cell line.

6. Generation of stable expression cell lines

4T1 *Enpp1*$^{-/-}$ cells generated above were then virally transfected to stably express WT or T238A mouse ENPP1, giving rise to ENPP1$^{WT\text{-}OE}$ or ENPP1$^{T238A\text{-}OE}$ cell lines. Briefly, lentiviral packaging plasmids (pHDM-G, pHDM-Hgmp2, pHDM-tat1b, and pRC.CMV-rev1b) were purchased from Harvard Medical School. 500 ng of pLenti-CMV-mENPP1-GFP-Puro, pLenti-CMV-mENPP1-T238A-GFP-Puro, or pLenti-CMV-mENPP1-A84S-GFP-Puro plasmid, and 500 ng of each of the packaging plasmids were transfected into 293T cells using FuGENE 6 transfection reagent (Promega). The viral media was exchanged after 24 h, harvested after 48 h and passed through a 0.45 µm filter, and used to transduce 4T1 *Enpp1*$^{-/-}$ cells (4T1 cells are used as 4T1-luc cells already carry puromycin resistance which will interfere with subsequent drug selection process). 48 hours later, cells were selected with 1–2 µg/ml puromycin, single-cell cloned, and 4-6 clones were pooled after verification by western blot and activity assay. E0771.lmb cells were virally transduced with empty pLenti-TetONE-FLAG-Puro vector to stably carry puromycin resistance following the previously described lentiviral transfection protocol, giving rise to the E0771.LMB.PuroR cell line used in metastatic models.

7. Serum and lysate preparation

Mouse blood was collected through terminal cardiac puncture and spun at 2,000 x g for 5 min. The resulting serum layer was collected and stored at -80°C until use. Cell lysate preparation for cGAMP degradation activity assay is as following: cells from a confluent well in a 6 well plate was collected in 1 mL of PBS, centrifuged at 1000 x *g* for 3 minutes, lysed in 50-100 µL of lysis buffer (10 mM Tris pH 9, 150 mM NaCl, 10 µM $ZnCl_2$, 1% NP-40), and stored in -20°C until use. For western blotting, cells were lysed on the plate in 100-250 µL of Laemmli sample buffer, boiled at 95°C for 5 minutes and sonicated. Mouse tumor lysate (100 mg/mL) for cGAMP degradation activity assay were generated by lysing tissues in 10 mM Tris pH 7.5, 150 mM NaCl, 10 µM $ZnCl_2$, 1.5% NP-40, and freshly added protease inhibitors (cOmplete, EDTA-free protease inhibitor cocktail, Sigma-Aldrich). Organ lysates were then homogenized with a bead homogenizer (Omni International) and stored at -20°C until use.

8. Western blotting

Cell lysates were separated on an SDS-polyacrylamide gel (Genscript) and transferred to a nitrocellulose membrane using a wet transfer system (BioRad). Primary antibody mouse anti-tubulin (1:2000) and rabbit anti-GFP (1:1000) were purchased from Cell Signaling and added overnight at 4°C, followed by three washes in TBS-T (1x TBS-0.1% tween). Secondary antibody IRDye 800CW goat anti-rabbit (1:15,000) and IRDye 680RD goat anti-mouse (1:15,000) were purchased from Li-COR Biosciences and added for 1 h at room temperature, followed by three additional washes in TBS-T. Blots were imaged in IR using a LI-COR Odyssey Blot Imager. Bands were quantified using ImageJ.

9. Quantification and statistical analysis

In cGAMP degradation assays, half-life was obtained by one phase exponential decay fitting with Prism software, with intercept (Y0) set up to be the initial cGAMP concentration, and plateau set to be 0. In proliferation assays, proliferation rate was obtained by exponential fitting with Prism software. All statistical tests were performed using GraphPad Prism software and are noted in the figure legends. Data are presented as the mean ± standard deviation unless otherwise stated.

Table S1. Reagent or Resources

REAGENT or RESOURCE	SOURCE	IDENTIFIER
Antibodies		
Alexa Fluor 594 anti-CD8a (53-6.7)	BioLegend	Cat# 100758
Alexa Fluor 700 anti-CD45 (30-F11)	BioLegend	Cat# 103128
APC anti-F4/80 (BM8)	BioLegend	Cat# 123116
APC anti-FoxP3 (3G3)	Tonbo Biosciences	Cat# 20-5773
Brilliant Violet 421 anti-CD11b (M1/70)	BioLegend	Cat# 101251
Brilliant Violet 510 anti-F4/80 (BM8)	BioLegend	Cat# 123135
Brilliant Violet 570 anti-Ly-6C (HK1.4)	BioLegend	Cat# 128030
Brilliant Violet 650 anti-CD45 (30-F11)	BioLegend	Cat# 103151
Brilliant Violet 650 anti-CD206 (C068C2)	BioLegend	Cat# 141723
Brilliant Violet 785 anti-CD8a (53-6.7)	BioLegend	Cat# 100749
Brilliant Violet 785 anti-CD11c (N418)	BioLegend	Cat# 117335
Brilliant Violet 785 anti-CD62L (MEL-14)	BioLegend	Cat# 104440
BUV 395 anti-CD103 (M290)	BD Biosciences	Cat# 740238
BUV 563 anti-Ly-6G (1A8)	BD Biosciences	Cat# 612921
BUV 805 anti-CD4 (GK1.5)	BD Biosciences	Cat# 612900
eFluor 450 anti-CD25 (PC61.5)	eBioscience Invitrogen	Cat# 48-0251-82
FITC anti-I-A/I-E (M5/114.15.2)	BioLegend	Cat# 107606
PE anti-CD11c (N418)	BioLegend	Cat# 117308
PerCP-Cy5.5 anti-I-A/I-E (M5/114.15.2)	BioLegend	Cat# 107626
PerCP-eFluor 710 anti-CD3e (eBio500A2)	eBioscience Invitrogen	Cat# 46-0033-82
TruStain FcX (anti-CD16/32)	BioLegend	Cat# 101320
Rabbit anti-phospho-IRF-3 (Ser396) (D6O1M)	Cell Signaling	Cat# 10327
VioGreen anti-Ly-6C (1G7.G10)	Miltenyi Biotec	Cat# 130-102-207
Rabbit anti-GFP (D5.1)	Cell Signaling	Cat# 2956
Mouse anti-tubulin (DM1A)	Cell Signaling	Cat# 3873
IRDye 800CW goat anti-rabbit	LI-COR Biosciences	Cat# 926-32211
IRDye 680RD goat anti-mouse	LI-COR Biosciences	Cat# 926-68070
Chemicals, peptides, and recombinant proteins		
DMEM	Corning Cellgro	Cat# MT10013CV
RPMI 1640	Corning Cellgro	Cat# 10-040-CV
IMDM, powder	ThermoFisher	Cat# 1220069
FBS	R&D Systems	Cat# S11150
Heat-inactivated FBS	R&D Systems	Cat# S11150H
Penicillin/streptomycin	ThermoFisher	Cat# 15140163
HEPES (1M)	Gibco	Cat# 15-630-080
2’3’-cGAMP	This paper	N/A
[^{32}P] cGAMP	This paper	N/A
Recombinant sscGAS	(Ritchie *et al.*, 2019)	N/A
[α-^{32}P] ATP, 250 µCi	PerkinElmer	Cat# BLU003H250UC
GTP, disodium salt hydrate	Sigma Millipore	Cat# G8877-1G
Herring testes DNA	Sigma Millipore	Cat# D6898-1G
HP-TLC silica gel aluminum	EMD Millipore	Cat# 1.05548.0001
Nonident P40 substitute (NP40)	Sigma-Aldrich	Cat# 74385

cOmplete, EDTA-free protease inhibitor Cocktail	Sigma-Aldrich	Cat# 04693132001
FuGENE 6	Promega	Cat# E2691
Neutralizing STING (WT)	(Carozza, Böhnert, *et al.*, 2020)	N/A
Non-binding STING (R237A)	(Carozza, Böhnert, *et al.*, 2020)	N/A
Collagenase from Clostridium histolyticum	Sigma-Aldrich	Cat# 5138
6-Thioguanine, $\geq$ 98%	Sigma-Aldrich	Cat# A4882
Methylene blue	Sigma-Aldrich	Cat# M9140
Critical commercial assays		
Live/Dead fixable near-IR dead cell stain kit	ThermoFisher	Cat# L10119
Live/Dead fixable blue dead cell stain kit	ThermoFisher	Cat# L23105
High sensitivity D1000 ScreenTape	Agilent	Cat# 5067-5584
High sensitivity D1000 Reagents	Agilent	Cat# 5067-5585
High sensitivity D5000 ScreenTape	Agilent	Cat# 5067-5592
High sensitivity D5000 Reagents	Agilent	Cat# 5067-5593
Live/Dead fixable blue dead cell stain kit	ThermoFisher	Cat# L23105
Chromium Next GEM Single Cell 3' HT Reagent Kits v3.1 (Dual Index)	10X Genomics	Cat# CG000422
NextSeq 2000 P3 Reagent (100 Cycles)	Illumina	Cat# 20040559
Deposited data		
scRNA-seq raw files	This paper	GSE233659
scRNA-seq processed files	This paper	GSE233659
METABRIC patient dataset	(Curtis *et al.*, 2012)	EGAS00000000083 (EGA, http://www.ebi.ac.uk/ega/)
IPSY-2 patient dataset	(Wolf *et al.*, 2022)	GSE194040
Experimental models: Cell lines		
Human: 293T cGAS *ENPP1*$^{-/-}$ cells	(Carozza, Böhnert, *et al.*, 2020)	N/A
Mouse: E0771 cells	CH3 BioSystems	Cat# 94001, RRID: CVCL_GR23
Mouse: E0771.lmb cells	ATCC	Cat# CRL-3405
Mouse: E0771.lmb.PuroR cells	This paper	N/A
Mouse: 4T1-luciferase (4T1-luc) cells	(Vilalta *et al.*, 2014)	N/A
Mouse: 4T1-luc *Enpp1*$^{-/-}$ cells	(Carozza, Böhnert, *et al.*, 2020)	N/A
Mouse: 4T1 cells	ATCC	Cat# CRL-2539, RRID: CVCL_0125
Mouse: 4T1 *Enpp1*$^{-/-}$ cells	This paper	N/A
Mouse: 4T1 ENPP1^{WT-OE} cells	This paper	N/A
Mouse: 4T1 ENPP$^{T238A-OE}$ cells	This paper	N/A
Experimental models: Organisms/strains		
Mouse: C57BL/6J	The Jackson Laboratory	JAX: 000664
Mouse: BALB/cJ	The Jackson Laboratory	JAX: 000651
Mouse: C57BL/6J-*Sting*^{gt}/*J*	The Jackson Laboratory	JAX: 017537

Mouse: C57BL/6J-*Enpp1*$^{asj/GrsrJ}$	The Jackson Laboratory	JAX: 012810
Mouse: BALB/cJ-*Enpp1*$^{asj-2J/GrsrJ}$	The Jackson Laboratory	JAX: 019107
Mouse: C57BL/6J-*Enpp1*^{H362A}	(Carozza *et al.*, 2022)	N/A
Mouse: C57BL/6J-*Enpp1*^{H362A} x *Sting*$^{-/-}$	(Carozza *et al.*, 2022)	N/A
Mouse: FVB/N-Tg(MMTV-PyVT) 634Mul/J	The Jackson Laboratory	JAX: 002374
Mouse: B6;FVB-MMTV	This paper	N/A
Mouse: B6;FVB-*Enpp1*^{H362A} x MMTV	This paper	N/A
Oligonucleotides		
See Table S1 for PCR primers	This paper	N/A
See Table S1 for *Enpp1* sgRNAs	(Carozza *et al.*, 2022)	N/A
Recombinant DNA		
pcDNA3-mENPP1-Flag	Genscript	N/A
pcDNA3-mENPP1-T238A-Flag	This paper	N/A
pcDNA3-hENPP1-Flag	Genscript	N/A
pcDNA3-hENPP1-K173Q-Flag	This paper	N/A
pLenti-CMV-GFP-Puro	Addgene	RRID: Addgene_17448
pLenti-CMV-mENPP1-GFP-Puro	This paper	N/A
pLenti-CMV-mENPP1-T238A-GFP-Puro	This paper	N/A
PX458	Addgene	RRID: Addgene_48138
Software and algorithms		
Prism 9.1.0	Graphpad	https://www.graphpad.com/scientific-software/prism/
ImageJ 2.0.0	(Schneider, Rasband and Eliceiri, 2012)	https://imagej.nih.gov/ij/
Pymol		https://www.pymol.org/2/
FlowJo V10	FlowJo, LLC	https://www.flowjo.com/
Cell Ranger (version 3.1.0.)	10x Genomics	https://support.10xgenomics.com/single-cell-gene-expression/software/overview/welcome
Cellenics associated with scRNA-seq	Cellenics	https://scp.biomage.net/data-management

Table S1. Oligonucleotide Sequences

Name	Sequence (5'->3')
Primers for cloning	
mENPP1_T238A_fwd	GCCTATGTACCCTACCAAGgcgTTTCCCAATCATTACAGC
mENPP1_T238A_rev	GCTGTAATGATTGGGAAAcgcCTTGGTAGGGTACATAGGC
mENPP1_seq_fwd	CTACAGTTCTGTGTGCCAAG
mENPP1_seq_rev	CCATTATTGGGAGCTGGGATCAAACC
pLenti_mENPP1_fwd	gccatccacgctgttttgacctccatagaagacaccgactctagaGATCCGCCACCATGGAGC
pLenti_mENPP1_rev	aacagctcctcgcccttgctcaccatggtggcgaccggtggatccCAGAATTCGTCTTCTTGGCTGAAGATTG
For CRISPR editing cells	
mENPP1_sgRNA1_for	caccgGCTCGCGCCCATGGACCT
mENPP1_sgRNA1_rev	aaacAGGTCCATGGGCGCGAGCc
mENPP1_sgRNA2_for	caccgATATGACTGTACCCTACGGG

mENPP1_sgRNA2_rev	aaacCCCGTAGGGTACAGTCATATc
mENPP1_sgRNA3_for	caccgGTGACCTGCAGGGTCCTT
mENPP1_sgRNA3_rev	aaacAAGGACCCTGCAGGTCACC
mENPP1_sgRNA4_for	caccgTGTGTAGCTGTGACACTCAG
mENPP1_sgRNA4_rev	aaacCTGAGTGTCACAGCTACACA
mENPP1_sgRNA12_seq_fwd	GCCAAATACCCGGGGCGTTG
mENPP1_sgRNA12_seq_rev	AAATCGGCGTCCTGTTTCCAGAAG
mENPP1_sgRNA34_seq_fwd	GGGTAGTGTGACAATAATTTATGG
mENPP1_sgRNA34_seq_rev	CAGGAGGGGGATATAAATGCC

References

1. Ritchie, C. *et al.* (2019) 'SLC19A1 Is an Importer of the Immunotransmitter cGAMP', *Molecular Cell*. Elsevier Inc., 75(2), pp. 372-381.e5. doi: 10.1016/j.molcel.2019.05.006.

2. Carozza, J. A., Böhnert, V., *et al.* (2020) 'Extracellular cGAMP is a cancer-cell-produced immunotransmitter involved in radiation-induced anticancer immunity', *Nature Cancer*. Springer US, 1(2), pp. 184–196. doi: 10.1038/s43018-020-0028-4.

3. Curtis, C. *et al.* (2012) 'The genomic and transcriptomic architecture of 2,000 breast tumours reveals novel subgroups', *Nature 2012 486:7403*. Nature Publishing Group, 486(7403), pp. 346–352. doi: 10.1038/nature10983.

4. Wolf, D. M. *et al.* (2022) 'Redefining breast cancer subtypes to guide treatment prioritization and maximize response: Predictive biomarkers across 10 cancer therapies', *Cancer Cell*, 40(6), pp. 609-623.e6. doi: 10.1016/j.ccell.2022.05.005.

5. Vilalta, M. *et al.* (2014) 'Recruitment of circulating breast cancer cells is stimulated by radiotherapy', *Cell reports*. Cell Rep, 8(2), pp. 402–409. doi: 10.1016/J.CELREP.2014.06.011.

6. Carozza, J. A. *et al.* (2022) 'ENPP1's regulation of extracellular cGAMP is a ubiquitous mechanism of attenuating STING signaling', *Proceedings of the National Academy of Sciences of the United States of America*, 119(21), pp. 1–11. doi: 10.1073/pnas.2119189119.

7. Schneider, C. A., Rasband, W. S. and Eliceiri, K. W. (2012) 'NIH Image to ImageJ: 25 years of image analysis', *Nature Methods 2012 9:7*. Nature Publishing Group, 9(7), pp. 671–675. doi: 10.1038/nmeth.2089.
